# Effectiveness of Lung Cancer Screening by Sex and Tumor Histology: Extended, Pooled Analysis of the ITALUNG and LUSI Trials, with Comparison to Findings in the NLST

**DOI:** 10.34133/cancomm.0011

**Published:** 2026-02-24

**Authors:** Rudolf Kaaks, Francisco Omar Cortés-Ibáñez, Stefan Delorme, Erna Motsch, Verena Katzke, Claus-Peter Heussel, Hans-Ulrich Kauczor, Giulia Picozzi, Giuseppe Gorini, Francesca Maria Carozzi, Laura Carrozzi, Eugenio Paci, Donella Puliti, Mario Mascalchi

**Affiliations:** ^1^ Division of Cancer Epidemiology, German Cancer Research Center (DKFZ), Heidelberg, Germany.; ^2^ Translational Lung Research Center Heidelberg (TLRC-H), German Center for Lung Research (DZL), Heidelberg, Germany.; ^3^ Division of Radiology (E010), German Cancer Research Center (DKFZ), Heidelberg, Germany.; ^4^ Department of Diagnostic and Interventional Radiology with Nuclear Medicine, Thoraxklinik, Heidelberg University Hospital, Heidelberg, Germany.; ^5^ Department of Diagnostic and Interventional Radiology, University Hospital Heidelberg, Heidelberg, Germany.; ^6^ Clinical Epidemiology Unit, Institute for Cancer Research, Prevention, and Clinical Network (ISPRO), Florence, Italy.; ^7^ Pulmonary Unit, Cardiothoracic and Vascular Department, University Hospital of Pisa, Pisa, Italy.; ^8^ Research Coordination Unit, Meyer Children’s Hospital, Florence, Italy.; ^9^ Department of Clinical and Experimental Biomedical Sciences “Mario Serio”, University of Florence, Florence, Italy.

Randomized trials have shown that low-dose computed tomography (LDCT) screening can reduce lung cancer (LC)-related mortality, but questions remain about differences in screening effects by sex and/or tumor histology. The US National Lung Screening Trial (NLST) [1], the Dutch–Belgian Netherlands–Leuvens Longkanker Screenings Onderzoek study [2] and other randomized trials, including the German Lung Cancer Screening Intervention study (LUSI) [3] and the Italian Lung study (ITALUNG) [4], indicated a stronger LC-related mortality reduction in women than in men, although this heterogeneity was not statistically significant in any separate study. Only the NLST has, so far, examined the effects of LDCT screening on LC-related mortality by sex and histologic tumor subtype [1], showing an equal mortality reduction among men (risk ratio [RR] = 0.71) and women (RR = 0.70) for non-small-cell LC (NSCLC) of nonsquamous histology. For women only, NLST showed an additional, borderline significant mortality risk reduction (hazard ratio [HR] = 0.67; 95% confidence interval [CI]: 0.40 to 1.03) for small-cell LC (SCLC), which, however, was deemed a possible chance observation [1].

To further the understanding of how LDCT screening may affect mortality by sex and histology, we analyzed the combined data from the ITALUNG and LUSI trials (ITALUNG–LUSI). Findings were compared to, and additionally combined with, those from NLST. No trial participant was excluded. Detailed descriptions of trial participants, data collection, and statistical analyses are included in the Supplementary Materials and Tables S1 to S3. Lung tumor types were grouped into (a) lung adenocarcinomas (LUADs) with exclusion of tumors with lepidic/bronchioloalveolar growth pattern (formerly designated as bronchioloalveolar carcinomas [BACs]) (non-BAC LUAD); (b) nonmucinous BAC; (c) mucinous BAC; (d) lung squamous cell carcinomas (LUSCs); (e) other NSCLC including mostly NSCLC-not otherwise specified (NSCLC-NOS) and large-cell tumors; (f) SCLC; (g) other neuroendocrine tumors; and (h) tumors that were not histologically characterized (“unclassified”)

ITALUNG–LUSI and NLST showed remarkably similar patterns of screening-induced stage shifts and mortality reductions across tumor subtypes. In the ITALUNG–LUSI (Table S4) and NLST studies (Table S5), LDCT increased the diagnoses of LUAD, as well as other NSCLC (mostly NSCLC-NOS), with strong shifts toward earlier tumor stages. Non-BAC LUAD constituted the highest proportion of LDCT-detected tumors among both men (57.5% in ITALUNG–LUSI and 38.8% in NLST) and women (64.3% in ITALUNG–LUSI and 45.3% in NLST). Lepidic/nonmucinous BAC additionally represented 17.9% and 19.6% of LDCT-detected tumors in women (ITALUNG–LUSI and NLST, respectively) but only 5.5% (ITALUNG–LUSI) and 8.6% (NLST) in men. For LUSC, in both ITALUNG–LUSI and NLST, compared to the control arm, LDCT screening yielded an increase in stage I diagnoses, as well as a reduction in advanced-stage tumors, among men but not clearly so among women. For “other NSCLC”, the NLST showed that, compared with the control arm, LDCT screening resulted in more early-stage tumors and a greater reduction in advanced-stage tumors in both men and women, and it was also associated with fewer incident cases and fewer deaths. For SCLC, both the ITALUNG–LUSI and the NLST detected very few cases through screening, and the numbers of SCLC in the LDCT and control arms were comparable, with no increase in early-stage tumors through LDCT screening. This notwithstanding, in both the ITALUNG–LUSI and the NLST, the LDCT arms exhibited fewer SCLC incident cases and deaths among women but not among men.

Proportional hazard models showed reductions in overall LC-related mortality that were more pronounced for women than men in the ITALUNG–LUSI (HR = 0.52 [0.29 to 0.92] versus HR = 0.85 [0.63 to 1.14]), the NLST (HR = 0.77 [0.63 to 0.95] versus HR = 0.94 [0.81 to 1.10]), and the combined ITALUNG–LUSI and NLST analysis (HR = 0.74 [0.61 to 0.90] versus HR = 0.93 [0.80 to 1.06]; *P*_heterogeneity_ = 0.06) (Table 1). Histology-specific analyses indicated that women experienced a mortality reduction for SCLC in the ITALUNG–LUSI (8 years postscreening, HR = 0.27 [0.08 to 0.97]), the NLST (6 years postscreening, HR = 0.68 [0.45 to 1.02]), and the combined ITALUNG–LUSI and NLST analysis (6 years postscreening, HR = 0.63 [0.43 to 0.94]). For men, data showed no reduction in SCLC mortality (Table 1), and in the combined ITALUNG–LUSI and NLST data, the effects of LDCT screening on SCLC mortality differed significantly between the sexes (unadjusted for smoking, *P*_heterogeneity_ = 0.040). In NLST and in the trials combined, for the 2 sexes together, the relative hazards for mortality were also significantly reduced for non-BAC LUAD (in ITALUNG–LUSI plus NLST, HR = 0.80 [0.65 to 0.97], 6 years postscreening) and for “other” NSCLC (ITALUNG–LUSI plus NLST, HR = 0.66 [0.51 to 0.87], 6 years postscreening), and for these outcomes, there was no significant heterogeneity in HR estimates between the sexes. For LUSC, no mortality reduction was observed, overall or by sex. Sensitivity analyses of the combined ITALUNG–LUSI and NLST data showed no clear evidence for heterogeneity of effect for LDCT screening on LC-related mortality, overall or for histologic subtypes, by baseline smoking status (current versus former) or lifetime smoking duration (≤40 versus >40 years) (Tables S4 to S8).

**Table 1.** Screening-related HRs for lung cancer screening by sex, histology, and follow-up time after scheduled final screening in ITALUNG-LUSI, NLST, and the 3 trials combined.

Years of follow-up for mortality since scheduled final screening	Males + females				Males				Females			
	Screening (*n* = 3,642)	Control (*n* = 3,616)	Δ	HR (95% CI)^b^	Screening (*n* = 2,350)	Control (*n* = 2,346)	Δ	HR (95% CI)^b^	Screening (*n* = 1,292)	Control (*n* = 1,270)	Δ	HR (95% CI)^b^
ITALUNG–LUSI^a^												
*All subtypes* *^c^*												
**2**	41	52	−11	0.79 (0.52–1.19)	37	38	−1	0.95 (0.60–1.50)	4	14	−10	**0.36 (0.13–0.99)**
**4**	56	80	−24	**0.70 (0.50–0.98)**	49	59	−10	0.83 (0.56–1.21)	7	21	−14	**0.36 (0.16–0.81)**
**6**	78	106	−28	**0.72 (0.54–0.96)**	66	78	−12	0.85 (0.61–1.17)	12	28	−16	**0.42 (0.22–0.83)**
**8**	97	127	−30	**0.76 (0.58–0.98)**	79	93	−14	0.85 (0.63–1.14)	18	34	−16	**0.52 (0.29–0.92)**
*Non-BAC LUAD* *^d^*												
**2**	18	14	+4	1.20 (0.61–2.38)	16	8	+8	1.79 (0.79–4.04)	2	6	−4	0.33 (0.07–1.63)
**4**	23	25	−2	0.96 (0.55–1.67)	19	17	+2	1.25 (0.65–2.41)	4	8	−4	0.44 (0.14–1.43)
**6**	31	35	−4	0.83 (0.52–1.34)	25	25	0	0.93 (0.54–1.59)	6	10	−4	0.59 (0.22–1.63)
**8**	37	46	−9	0.82 (0.53–1.26)	30	34	−4	0.88 (0.54–1.44)	7	12	−5	0.60 (0.25–1.45)
*LUSC* *^e^*												
**2**	8	11	−3	0.71 (0.27–1.87)	7	9	−2	0.76 (0.26–2.20)	1	2	−1	0.50 (0.05–5.56)
**4**	11	19	−8	0.55 (0.26–1.15)	10	17	−7	0.53 (0.24–1.19)	1	2	−1	0.50 (0.05–5.56)
**6**	18	28	−10	0.64 (0.35–1.16)	15	24	−9	0.63 (0.33–1.19)	3	4	−1	0.74 (0.16–3.29)
**8**	21	30	−9	0.74 (0.43–1.29)	16	26	−10	0.64 (0.35–1.20)	5	4	+1	1.22 (0.33–4.56)
*Other NSCLC* *^f^*												
**2**	2	7	−5	0.28 (0.05–1.37)	2	4	−2	0.49 (0.09–2.72)	0	3	−3	NE
**4**	5	10	−5	0.49 (0.17–1.46)	4	7	−3	0.56 (0.16–1.94)	1	3	−2	0.33 (0.03–3.25)
**6**	6	11	−5	0.54 (0.20–1.47)	5	8	−3	0.62 (0.20–1.90)	1	3	−2	0.33 (0.03–3.25)
**8**	10	12	−2	0.82 (0.35–1.91)	8	8	0	1.00 (0.37–2.65)	2	4	−2	0.49 (0.09–2.70)
*SCLC* *^g^*												
**2**	11	17	−6	0.75 (0.35–1.58)	10	15	−5	0.78 (0.36–1.73)	1	2	−1	0.50 (0.04–5.58)
**4**	15	22	−7	0.68 (0.35–1.31)	14	16	−2	0.87 (0.43–1.79)	1	6	−5	0.17 (0.02–1.38)
**6**	20	28	−8	0.71 (0.40–1.26)	18	19	−1	0.95 (0.50–1.80)	2	9	−7	0.22 (0.05–1.02)
**8**	24	30	−6	0.80 (0.47–1.36)	21	19	+2	1.10 (0.59–2.05)	3	11	−8	**0.27 (0.08–0.97)**
Years of follow-up for mortality since scheduled final screening	Males + females				Males				Females			
	Screening(*n* = 26,722)	Control (*n* = 26,730)	Δ	HR (95% CI)^b^	Screening (*n* = 15,769)	Control (*n* = 15,761)	Δ	HR (95% CI)^b^	Screening (*n* = 10,953)	Control (*n* = 10,969)	Δ	HR (95% CI)^b^
NLST^h^												
*All subtypes* *^c^*												
**2**	230	271	−41	0.84 (0.71–1.01)	158	171	−13	0.92 (0.74–1.14)	72	100	−28	**0.72 (0.53–0.97)**
**4**	387	477	−90	**0.80 (0.70–0.92)**	260	293	−33	0.88 (0.74–1.04)	127	184	−57	**0.69 (0.55–0.86)**
**6**	497	561	−64	**0.88 (0.78–0.99)**	328	344	−16	0.94 (0.81–1.10)	169	217	−48	**0.77 (0.63–0.95)**
*Non-BAC LUAD* *^d^*												
**2**	66	94	−28	**0.69 (0.51–0.96)**	46	59	−13	0.77 (0.53–1.14)	20	35	−15	**0.57 (0.33–0.99)**
**4**	113	163	−50	**0.69 (0.54–0.87)**	70	102	−32	**0.68 (0.50–0.92)**	43	61	−18	0.70 (0.47–1.04)
**6**	154	196	−42	**0.78 (0.63–0.96)**	98	122	−24	0.80 (0.61–1.04)	56	74	−18	0.75 (0.43–1.06)
*LUSC* *^e^*												
**2**	57	38	+19	1.49 (0.99–2.25)	42	28	+14	1.49 (0.92–2.40)	15	10	+5	1.50 (0.67–3.33)
**4**	91	73	+18	1.15 (0.81–1.65)	70	54	+16	1.28 (0.90–1.83)	21	19	+2	1.04 (0.57–1.93)
**6**	111	87	+24	1.25 (0.95–1.66)	82	64	+18	1.27 (0.91–1.76)	29	23	+6	1.20 (0.70–2.07)
*Other NSCLC* *^f^*												
**2**	46	67	−21	**0.67 (0.46–0.98)**	27	44	−17	**0.61 (0.38–0.98)**	19	23	−4	0.78 (0.42–1.45)
**4**	68	113	−45	**0.59 (0.44–0.80)**	42	68	−26	**0.61 (0.42–0.90)**	26	45	−19	**0.57 (0.35–0.92)**
**6**	86	126	−40	**0.69 (0.52–0.90)**	52	75	−23	0.71 (0.49–1.01)	34	51	−17	0.66 (0.42–1.02)
*SCLC* *^g^*												
**2**	52	60	−8	0.86 (0.59–1.25)	36	33	+3	1.08 (0.67–1.74)	16	27	−11	0.59 (0.32–1.10)
**4**	87	103	−16	0.84 (0.63–1.11)	57	56	+1	1.01 (0.70–1.46)	30	47	−17	0.64 (0.40–1.00)
**6**	109	120	−11	0.90 (0.70–1.17)	71	64	+7	1.10 (0.78–1.54)	38	56	−18	0.68 (0.45–1.02)
Years of follow-up for mortality since scheduled final screening	Males + females				Males				Females			
	Screening (*n* = 30,364)	Control (*n* = 30,346)	Δ	HR (95% CI)^b^	Screening (*n* = 18,119)	Control (*n* = 18,107)	Δ	HR (95% CI)^b^	Screening (*n* = 12,245)	Control (*n* = 12,239)	**Δ**	HR (95% CI)^b^
ITALUNG–LUSI^a^ plus NLST^h^												
*All subtypes* *^c^*												
**2**	271	323	−52	**0.83 (0.71–0.98)**	195	209	−14	0.92 (0.76–1.12)	76	114	−38	**0.67 (0.50–0.90)**
**4**	443	557	−114	**0.79 (0.70–0.90)**	309	352	−43	0.87 (0.75–1.01)	134	205	−71	**0.65 (0.52–0.81)**
**6**	560	648	−88	**0.85 (0.76–0.96)**	380	406	−26	0.93 (0.80–1.06)	180	242	−62	**0.74 (0.61–0.90)**
*Non-BAC LUAD* *^d^*												
**2**	84	108	−24	0.76 (0.57–1.01)	62	67	−5	0.89 (0.63–1.26)	22	41	−19	**0.53 (0.32–0.90)**
**4**	135	187	−52	**0.71 (0.57–0.89)**	88	119	−31	**0.74 (0.56–0.97)**	47	68	−21	**0.67 (0.46–0.97)**
**6**	179	223	−44	**0.80 (0.65–0.97)**	118	140	−22	0.84 (0.65–1.07)	61	83	−22	0.73 (0.52–1.02)
*LUSC* *^e^*												
**2**	65	49	+16	1.33 (0.91–1.93)	49	37	+12	1.32 (0.86–2.04)	16	12	+4	1.33 (0.63–2.81)
**4**	102	92	+10	1.08 (0.81–1.43)	80	71	+9	1.09 (0.79–1.50)	22	21	+1	1.00 (0.58–1.87)
**6**	123	109	+14	1.12 (0.87–1.45)	92	83	+9	1.10 (0.82–1.48)	31	26	+5	1.19 (0.70–2.00)
*Other NSCLC* *^f^*												
**2**	48	74	−26	**0.65 (0.45–0.93)**	29	48	−19	**0.59 (0.37–0.93)**	19	26	−7	0.77 (0.43–1.38)
**4**	73	123	−50	**0.59 (0.44–0.79)**	46	75	−29	**0.61 (0.42–0.88)**	27	48	−21	**0.56 (0.35–0.90)**
**6**	91	136	−45	**0.66 (0.51–0.87)**	56	82	−26	**0.68 (0.48–0.95)**	35	54	−19	**0.64 (0.42–0.99)**
*SCLC* *^g^*												
**2**	63	77	−14	0.84 (0.60–1.17)	46	48	−2	1.00 (0.66–1.49)	17	29	−12	0.59 (0.32–1.06)
**4**	102	125	−23	0.81 (0.62–1.05)	71	72	−1	0.98 (0.70–1.36)	31	53	−22	**0.58 (0.37–0.91**)
**6**	126	143	−17	0.87 (0.69–1.11)	86	80	+6	1.07 (0.79–1.44)	40	63	−23	**0.63 (0.43–0.94**)

HR was estimated using the proportional hazards model. Bold figures indicate hazard rates that significantly differ from 1.00 (*P* < 0.05).

^a^ Analyses of ITALUNG–LUSI included lung cancer mortality endpoints up to 8 years after scheduled final screening, irrespective of the date of preceding lung cancer diagnosis.

^b^ Adjusted by age.

^c^ All subtypes: non-BAC LUAD^d^ (see below), LUSC^e^ (see below), other NSCLC^f^ (see below), and SCLC^g^ (see below); lepidic/nonmucinous BAC: ICD-O-3 = 8250 and 8252; mucinous BAC: ICD-O-3 = 8253 and 8254; other neuroendocrine: ICD-O-3 = 8240, 8246, and 8249; and unclassified tumors: ICD-O-3 = 8000 and 8001.

^d^ Adenocarcinomas without BAC: ICD-O-3 = 8140, 8230, 8255, 8260, 8310, 8480, 8490, 8550, and 8560.

^e^ Squamous cell carcinoma: ICD-O-3 = 8052, 8070, 8071, 8072, 8074, 8076, 8078, and 8083.

^f^ Other non-small cell carcinoma: ICD-O-3 = 8010,8012, 8013, 8020, 8021, 8022, 8032, 8033, 8046, 8050, and 8980.

^g^ Small cell carcinoma: ICD-O-3 = 8041, 8042, 8044, and 8045.

^h^ Analyses of data from NLST and of pooled ITALUNG–LUSI plus NLST (3-trial) data were restricted to lung cancer mortality end points with a corresponding diagnosis within maximally 4 years after last scheduled screening.

Δ, difference between screening and control arm; HR: hazard ratio; CI: confidence interval; LUSI: Lung Cancer Screening Intervention study; ITALUNG: Italian Lung Study; NLST: National Lung Screening Trial; BAC: bronchiolo-alveolar carcinoma; ICD-O-3: International Classification of Diseases for Oncology, Third edition; NE: not evaluable; LUAD: lung adenocarcinoma; LUSC: lung squamous cell carcinomas; NSCLC: non-small cell lung cancers; SCLC: small-cell lung cancer.

The reduced incidence and mortality by SCLC in the LDCT arms among women are paradoxical, as very few SCLC cases were screen-detected (and without a clear stage shift). A likely explanation for this paradox is the removal of early-stage tumors that present with a different histology but harbor precursor cells for the parallel or later development of SCLC (intratumor clonal heterogeneity and/or histologic transdifferentiation). A substantial proportion of lung tumors, including screen-detected tumors, show mixed histology or multiple (synchronous or meta-synchronous) primaries of diverse histology, often including a LUAD component [5]. Considerable evidence also indicates that SCLC can develop either along with histologically diverse tumor components or through histological transformation from NSCLC precursors, most commonly from LUAD [6,7]. Our observations (Table 1) indicate that the gap in SCLC incidence between the LDCT and control arms progressively widens with years since the final screening. A 2019 report from NLST [8], based on partially complete follow-up data up to 9 years after final screening (not released for external research), indicated that the deficit of incident SCLC cases in the LDCT arm of NLST had increased further. These observations suggest that precursor lesions might include slowly progressing, minimally invasive tumors at the time of LDCT detection.

Although the available ITALUNG–LUSI plus NLST trial data do not allow a direct answer as to why LDCT screening reduces SCLC incidence and mortality in women only, we speculate that this is related to a more frequent occurrence of slowly progressing, minimally invasive adenocarcinomas in women than men. We further hypothesize that the latter could include a high percentage of adenocarcinomas with aberrant epidermal growth factor receptor (EGFR) signaling [6]. EGFR mutations provide a driving oncogenic stimulus for LUAD development, independent of smoking [9], more often in women than in men and more often in Asian than Caucasian populations [9,10], and have also been related to transformation of LUAD to SCLC [6,7].

Overall, our analyses of the combined ITALUNG/LUSI plus NLST data indicate that LDCT screening could reduce mortality from LUAD and from “other NSCLC” in both sexes. Moreover, our findings from the ITALUNG–LUSI alone and in combination with those from the NLST showed an additional reduction in both the incidence and mortality from SCLC among women only. This sex-specific effect may reflect the early detection and removal of slowly progressive tumors that would eventually evolve into SCLC but initially present with different predominant histology and which may develop mostly in women.

The present study is limited by the small number of LC cases and deaths in the ITALUNG–LUSI cohort and the restricted follow-up duration in the NLST for histology-specific LC diagnosis. Therefore, our findings should be further validated in additional screening trials. Future studies should include molecular analyses of screen-detected versus nonscreen-detected tumors to provide further insight into the biological mechanisms underpinning sex-specific effects of LDCT screening.

## Ethical Approval

In all 3 trials (ITALUNG, LUSI, and NLST), participants provided informed consent. The ITALUNG trial was approved by the local ethical review boards of the Universities of Florence, Pistoia, and Pisa (approval numbers: 29-30 of 2003 September 30; 23 of 2003 October 27; and 00028543 of 2004 May 13). LUSI was approved by the ethical review board of Heidelberg University (073/2001). NLST obtained ethical approval from each of 33 participating institutions (see https://cdas.cancer.gov/learn/nlst/trial-summary/).

## Data Availability

R.K., F.O.C.-I., D.P., and M.M. have full access to all the data in the study and take responsibility for the integrity of the data and the accuracy of the data analysis. The data of ITALUNG and LUSI cannot be shared publicly because of limitations in the original informed consent by the trial participants, aiming to protect the privacy of individuals who participated in the study and their medical data. The data of the NLST study, used in the present analyses, are available upon request from the US National Cancer Institute (https://cdas.cancer.gov/datasets/nlst/)
