# Supplementary material for: Effectiveness of Lung Cancer Screening by Sex and Tumor Histology: Extended, Pooled Analysis of the ITALUNG and LUSI Trials, with Comparison to Findings in the NLST
Source: Cancer Commun (Lond). 2026 Feb 24;46:0011. doi: 10.34133/cancomm.0011 (PMC12929912; doi:10.34133/cancomm.0011)
Supplement: Supplementary 1 — Supplementary Methods Tables S1 to S8 [file cancomm.0011.f1.docx]

**Supplementary Materials**

**Effectiveness of lung cancer screening by sex and tumor histology: extended, pooled analysis of the ITALUNG and LUSI trials, with comparison to findings in the** **NLST**

Rudolf Kaaks^1, 2, †, *^, Francisco Omar Cortés-Ibáñez^1, 2, †^, Stefan Delorme^3^, Erna Motsch^1^, Verena Katzke^1^, Claus-Peter Heussel^2, 4, 5^, Hans-Ulrich Kauczor^2, 5^, Giulia Picozzi^6^, Giuseppe Gorini^6^, Francesca Maria Carozzi^6^, Laura Carrozzi^7^, Eugenio Paci^6^, Donella Puliti^6, 8^, Mario Mascalchi^1, 9, *^

1. Division of Cancer Epidemiology, German Cancer Research Center (DKFZ), Heidelberg, Germany.
2. Translational Lung Research Center Heidelberg (TLRC-H), German Center for Lung Research (DZL), Heidelberg, Germany.
3. Division of Radiology (E010), German Cancer Research Center (DKFZ), Heidelberg, Germany.
4. Department of Diagnostic and Interventional Radiology with Nuclear Medicine, Thoraxklinik, Heidelberg University Hospital, Heidelberg, Germany.
5. Department of Diagnostic and Interventional Radiology, University Hospital Heidelberg, Heidelberg, Germany.
6. Clinical Epidemiology Unit, Institute for Cancer Research, Prevention and Clinical Network (ISPRO), Florence, Italy.
7. Pulmonary Unit, Cardiothoracic and Vascular Department, University Hospital of Pisa, Italy.
8. Research Coordination Unit, Meyer Children’s Hospital, Florence, Italy.
9. Department of Clinical and Experimental Biomedical Sciences “Mario Serio”, University of Florence, Florence, Italy.

^†^These authors contributed equally

^*^Correspondence to:

Rudolf Kaaks, Division of Cancer Epidemiology, German Cancer Research Center (DKFZ), Heidelberg 69120, Germany; [r.kaaks@dkfz-heidelberg.de](mailto:r.kaaks@dkfz-heidelberg.de)

Mario Mascalchi, Department of Clinical and Experimental Biomedical Sciences “Mario Serio”, University of Florence, Florence 50134, Italy; [mario.mascalchi@unifi.it](mailto:mario.mascalchi@unifi.it)

**Supplementary Methods**

**Study populations and their eligibility criteria**

Study populations, eligibility criteria, and methods used for prospective ascertainment and coding of overall lung cancer (LC) incidence and cause-specific mortality endpoints have been described in detail earlier for ITALUNG [1, 2], LUSI [3-5] and NLST [6, 7].

For ITALUNG [1, 2], study participants were recruited by an invitation letter, starting from patient lists of 269 general practitioners in three districts in the region of Tuscany. Besides age limits of 55-69 years, eligibility criteria were a history of ≥20 pack-years of cumulative smoking exposure and < 10 years since quitting for ex-smokers. For LUSI [3-5], participants were also recruited by invitation letters, but starting from population registers of the city of Heidelberg and the surrounding communes. Further to age limits of 50-69 years, eligibility criteria for LUSI included a smoking history of ≥15 cigarettes per day for ≥25 years, or alternatively of ≥10 cigarettes per day for ≥30 years, and <10 years since quitting for ex-smokers. In both trials, participants were randomized 1:1 to a computed tomography (CT) screening arm or a control arm without screening. Screening arm participants were offered 4 annual screenings in ITALUNG, performed between 2004 and 2010, and 5 annual screenings in LUSI, between 2007 and 2015. Both trials offered smoking cessation counseling to the participants of either arm, as described previously in greater detail [8, 9].

For the NLST [6, 7], participants were recruited from 33 medical centers across the United States of America (USA), and had to be 55-74 years of age with a cumulative smoking history of ≥30 pack-years and <15 years’ quitting time for ex-smokers. Individuals were randomly assigned 1:1 to either a low-dose computed tomography (LDCT) screening arm or a control arm in which a standard chest X-ray (CXR) was offered as an alternative screening tool. Both arms were offered up to 3 annual screenings, which took place from August 2002 to September 2007.

**Criteria for positive screening tests and further diagnostic investigations**

LUSI: Nodules first-time detected by LDCT, in any screening round, were classified by size (largest diameter) in four categories: (i) no nodules or less than 5 mm, (ii) 5–7 mm, (iii) 8–10 mm, and (iv) 10 mm or larger. Accordingly, after a two-step image evaluation and decision procedure by a trained radiologist and a senior radiologist, screening participants were (i) returned to regular annual screening, invited for earlier follow-up LDCT after (ii) 6 months or (iii) 3 months or (iv) recommended immediate diagnostic work-up. In screening rounds 2–5 (“incidence” screens), work-up of the nodules already observed in earlier screens was based exclusively on nodule growth, and classified in three categories: (i) no growth or volume doubling time (VDT) more than 600 days (returned to regular annual screening), (ii) doubling time within 400–600 days (invited for LDCT after 6 months) or (iii) doubling time 400 days or less (recommended immediate workup). Further details presented by Becker et al. [5].

ITALUNG: Positivity of the LDCT screening test was fundamentally based on the nodule size or growth measured in terms of mean diameter. In particular, the size threshold at baseline LDCT was 5 mm for non-calcified solid nodules and 10 mm for non-solid nodules, whereas significant growth was defined as an increase of at least 1 mm in the mean diameter of a solid nodule, or the appearance or increase of a solid component in a nonsolid or part-solid nodule in two successive LDCT examinations. At annual repeat screening rounds, the LDCT examination was considered positive if either a new solid, part-solid or nonsolid nodule was identified, or at least one solid, part-solid or nonsolid nodule already present in the last LDCT showed interim growth. If the new nodule had a mean diameter of 3 mm or less, the subject received a 6-month follow-up LDCT, whereas a 3-month follow-up LDCT was obtained in case of a new nodule with a mean diameter between 3mm and 5mm. In case of a nodule that was 5 mm or more in size, or if the screening test revealed multiple focal solid or nonsolid abnormalities consistent with inflammatory disease, a follow-up LDCT after 1 month of antibiotic therapy was recommended. In case of complete resolution of the abnormalities, the subject was sent for annual repeat screening, whereas, a further follow-up LDCT after 2 months was performed in case of partial or lack of resolution after antibiotic therapy. Further details presented by Lopes-Pegna et al. [1] and in Lopes-Pegna et al. [10].

When a solid nodule observed at baseline or repeat screening round attained a mean diameter of 8 mm or more, and persisted after antibiotic therapy, chest Fluorodeoxyglucose Positron Emission Tomography (FDG-PET) examination was recommended. However, in some cases of large lesions, strongly suggestive of malignancy, CT-guided FNAB (Fine Needle Aspiration Biopsy) or Fibro-Broncho-Scopy (FBS) was directly performed. For FDG-PET-positive nodules, a CT-guided FNAB with Rapid On Site Examination (ROSE) was recommended, whereas, a further 3-month follow-up LDCT was obtained in FDG-PET-indeterminate or -negative nodules. A 3-month follow-up LDCT was also scheduled for nodules with positive or indeterminate FDG-PET and negative or inconclusive CT-guided FNAB. All subjects showing no nodule growth at this latter follow-up LDCT were invited to the subsequent annual repeat LDCT scan. For pure nonsolid nodules of at least 10 mm diameter at baseline, and for new or growing nonsolid or part-solid nodules of at least 8 mm diameter at annual repeat screening, which persisted after antibiotic therapy, CT-guided FNAB was scheduled because FDG-PET is not indicated.

NLST: Low-dose CT scans that revealed any non-calcified nodule measuring at least 4 mm in any diameter and radiographic images that revealed any non-calcified nodule or mass were classified as positive, “suspicious for” lung cancer. Other abnormalities, such as adenopathy or effusion, could be classified as a positive result as well. At the third round of screening (T2), abnormalities suspicious for lung cancer that were stable across the three rounds could, according to the protocol, be classified as minor abnormalities rather than positive results. Further details are presented by Aberle et al. [11].

**Prospective follow-up for LC incidence and mortality**

In ITALUNG and LUSI, prospective ascertainment for lung cancer incidence (further to screen-detection) was based on record linkages with cancer registries (ITALUNG, LUSI), of annual self-reports followed by retrieval of medical records (LUSI). Vital status and cause-specific mortality were ascertained through linkages with mortality registers (ITALUNG, LUSI). For all deaths, medical records were collected for neutral (re-)evaluation of LC as the likely primary cause, by clinical expert panels. Follow-up for vital status evaluation of cause of death was performed until 31 December 2020 in ITALUNG, and until 30 April 2023 in LUSI. For incident lung cancer diagnoses, with histology information, follow-up was performed till 31 December 2019 in ITALUNG and till 30 April 2023 in LUSI. Our present analyses of ITALUNG-LUSI data, however, were restricted to 11 years after baseline randomization for ITALUNG and 12 years after randomization for LUSI, corresponding to a maximum of 8 years after last scheduled screening in both trials, which for both trials was the time where the ascertainment of incidence, mortality and histology were systematically retrieved and had reached maximum completeness. Within these follow-up time restrictions, in ITALUNG there were 94 LC diagnoses in the control arm (24 females, 70 males) and 87 in the screening arm (17 females, 70 males); in LUSI were diagnosed 96 LC cases in the control arm (33 females, 63 males) and 103 in the screening arm (31 females, 72 males). Accordingly, lung cancer-specific mortality cases in ITALUNG were 66 in the control arm (16 females, 50 males) and 53 in the screening arm (10 females and 43 males); in LUSI there were 61 in the control arm (18 females, 43 males) and 44 in the screening arm (8 females, 36 males).

In the NLST, as reported previously [7], prospective ascertainment of LC diagnoses was performed through regular contact with study participants (“active” follow-up) with extraction of medical records for participants with positive screen test or indication of LC diagnosis. Active follow-up lasted till December 31, 2009, corresponding to a median follow-up time of 6.5 (interquartile range [IQR] 6.1-6.9) years after randomization (4.5 [IQR 4.1-4.9] years after scheduled final screening). During this active follow-up period, vital status was assessed through regular questionnaire contacts plus linkages to the National Health Index (NDI). Data collection for LC histology was complete only for this active follow-up period. After 2009, participants were followed only passively through state cancer registries and the NDI [until 31 December 2015], but with information on tumor histology for only part of the study participants and without independent re-evaluation of whether LC was the likely primary cause of death.

In all trials, histological tumor classifications for incident LC cases were coded according to ICD-O-3. For statistical analyses, tumor types were grouped into 7 main categories including: 1) adenocarcinomas (LUAD) (without tumors lepidic/bronchioalveolar growth); 2) non-mucinous and 3) mucinous subgroups of tumors with lepidic/bronchiolo-alveolar growth pattern (formerly designated as bronchiolo-alveolar cancer, BAC); 4) squamous cell cancer (LUSC); 5) “other” non-small cell cancers (“other NSCLC”; including NSCLC not otherwise specified [NOS], unidentified carcinomas and large cell tumors); 6) small cell cancer (SCLC); 7) other neuro-endocrine tumors; and 8) tumors that were not histologically characterized (“unclassified”) [12-14]. Details of this grouping, with total numbers of observations in screening and control/CXR arms of ITALUNG-LUSI and NLST, are in the **Supplementary Table S1** below. For ITALUNG-LUSI, a re-ascertainment and retrieval of further clinical records in both trials allowed to have updated classification for cases previously reported as “unclassified histology.”

**Statistical analyses**

For the present analyses, we used complete follow-up data on LC incidence and mortality, overall and by histologic subtype, up to 11 years after randomization for ITALUNG and 12 years post-randomization for LUSI (i.e., up to 8 years after scheduled final screening in both trials). For NLST, as detailed above, complete data on tumor histology systematically collected only up to about 6 years after randomization (i.e., 4 years after scheduled final screening) and thereafter only for subsets of participants [7], whereas mortality data could be analyzed up to 8 years after randomization (i.e., up to 6 years after scheduled final screening in ITALUNG-LUSI and NLST).

For ITALUNG-LUSI, the present analyses included a total of 3,652 participants allocated to the combined screening arms (1,613 in ITALUNG, of which 1,406 actually took part in active screening; 2,029 in LUSI) and 3,616 participants allocated to the control arms (1,593 in ITALUNG; 2,023 in LUSI). NLST included a total of 26,722 participants in the LDCT arm, and 26,730 in the CXR control arm.

Basic tabulations were used to compare the number of incident LC cases and LC-related deaths by tumor histology and stage of diagnosis, in LDCT vs. control arms. Relative risks for LC-related mortality, overall and by subtype, were estimated by proportional hazards models adjusting for age at randomization and study. To ascertain the time point at which the greatest absolute and/or relative mortality reduction was reached, calculations were made for 2, 4, 6 and (for ITALUNG-LUSI only) 8 years after the scheduled final screening.

We additionally analyzed data for LC-related deaths that occurred among participants who had received an LC diagnosis within maximally 4 years after scheduled final screening (i.e., diagnosed up to 6, 7 or 8 years after randomization in NLST, ITALUNG and LUSI, respectively). This latter endpoint definition aligns with the diagnostic data from NLST made available by the US National Cancer Institute (NCI). Furthermore, this endpoint definition corresponds to that used in previous NLST analyses [7] to minimize dilution biases in estimated risk ratios for LC-related mortality, caused by progressively rising numbers of LC patients diagnosed after final screening in the LDCT arm who could not have benefited from early screen-detection within average LC detection lead times [15]. To compare sex- and histology-specific stage shifts and mortality reduction patterns, we performed parallel analyses of NLST data, plus analyses of ITALUNG-LUSI plus NLST combined. Statistical interaction terms were used to test whether the relative hazards of LC-related mortality varied significantly by sex or smoking history.

**References**

1. Lopes Pegna A, Picozzi G, Mascalchi M, Maria Carozzi F, Carrozzi L, Comin C, et al. Design, recruitment and baseline results of the ITALUNG trial for lung cancer screening with low-dose CT. Lung Cancer. 2009;64(1):34-40.

2. Paci E, Puliti D, Lopes Pegna A, Carrozzi L, Picozzi G, Falaschi F, et al. Mortality, survival and incidence rates in the ITALUNG randomised lung cancer screening trial. Thorax. 2017;72(9):825-31.

3. Becker N, Motsch E, Gross ML, Eigentopf A, Heussel CP, Dienemann H, et al. Randomized study on early detection of lung cancer with MSCT in Germany: study design and results of the first screening round. J Cancer Res Clin Oncol. 2012;138(9):1475-86.

4. Becker N, Motsch E, Gross ML, Eigentopf A, Heussel CP, Dienemann H, et al. Randomized Study on Early Detection of Lung Cancer with MSCT in Germany: Results of the First 3 Years of Follow-up After Randomization. J Thorac Oncol. 2015;10(6):890-6.

5. Becker N, Motsch E, Trotter A, Heussel CP, Dienemann H, Schnabel PA, et al. Lung cancer mortality reduction by LDCT screening-Results from the randomized German LUSI trial. Int J Cancer. 2020;146(6):1503-13.

6. Aberle DR, Adams AM, Berg CD, Black WC, Clapp JD, Fagerstrom RM, et al. Reduced lung-cancer mortality with low-dose computed tomographic screening. N Engl J Med. 2011;365(5):395-409.

7. National Lung Screening Trial Research T. Lung Cancer Incidence and Mortality with Extended Follow-up in the National Lung Screening Trial. J Thorac Oncol. 2019;14(10):1732-42.

8. Pistelli F, Aquilini F, Falaschi F, Puliti D, Ocello C, Lopes Pegna A, et al. Smoking cessation in the ITALUNG lung cancer screening: what does "teachable moment" mean? Nicotine Tob Res. 2019.

9. Bade M, Bahr V, Brandt U, Eigentopf A, Bruchert T, Gross ML, et al. Effect of smoking cessation counseling within a randomised study on early detection of lung cancer in Germany. J Cancer Res Clin Oncol. 2016;142(5):959-68.

10. Lopes Pegna A, Picozzi G, Falaschi F, Carrozzi L, Falchini M, Carozzi FM, et al. Four-year results of low-dose CT screening and nodule management in the ITALUNG trial. J Thorac Oncol. 2013;8(7):866-75.

11. Aberle DR, DeMello S, Berg CD, Black WC, Brewer B, Church TR, et al. Results of the two incidence screenings in the National Lung Screening Trial. N Engl J Med. 2013;369(10):920-31.

12. Travis WD, Brambilla E, Nicholson AG, Yatabe Y, Austin JHM, Beasley MB, et al. The 2015 World Health Organization Classification of Lung Tumors: Impact of Genetic, Clinical and Radiologic Advances Since the 2004 Classification. J Thorac Oncol. 2015;10(9):1243-60.

13. Travis WD, Brambilla E, Noguchi M, Nicholson AG, Geisinger KR, Yatabe Y, et al. International association for the study of lung cancer/american thoracic society/european respiratory society international multidisciplinary classification of lung adenocarcinoma. J Thorac Oncol. 2011;6(2):244-85.

14. Okudela K, Matsumura M, Arai H, Woo T. The nonsmokers' and smokers' pathways in lung adenocarcinoma: Histological progression and molecular bases. Cancer Sci. 2021;112(9):3411-8.

15. Hanley JA. Analysis of mortality data from cancer screening studies: looking in the right window. Epidemiology. 2005;16(6):786-90.

**Supplementary Table S1. Incident lung cancer cases in ITALUNG-LUSI and NLST by arm, by ICD-O-3 code, and their grouping for statistical analyses.**

| **ICD-O-3 code** | **Histological type** | **Incidence** | | | |
| --- | --- | --- | --- | --- | --- |
|  |  | **ITALUNG-LUSI** | | **NLST** | |
|  |  | Screening arm | Control Arm | Screening arm | Control Arm |
| 1. **Non-BAC lung adenocarcinoma** | | | | | |
| 8140/3 | Adenocarcinoma, NOS | 45 | 59 | 334 | 286 |
| 8230/3 | Solid carcinoma, NOS | 0 | 0 | 0 | 0 |
| 8255/3 | Adenocarcinoma with mixed subtypes | 22 | 9 | 6 | 3 |
| 8260/3 | Papillary adenocarcinoma, NOS | 9 | 3 | 8 | 5 |
| 8310/3 | Clear cell adenocarcinoma, NOS | 2 | 0 | 0 | 1 |
| 8323/3 | Mixed cell adenocarcinoma | 0 | 0 | 2 | 0 |
| 8480/3 | Mucinous adenocarcinoma | 2 | 1 | 4 | 2 |
| 8481/3 | Mucing-producing adenocarcinoma | 0 | 0 | 3 | 4 |
| 8490/3 | Signet ring cell carcinoma | 0 | 1 | 2 | 3 |
| 8550/3 | Acinar cell carcinoma | 13 | 4 | 6 | 8 |
| 8560/3 | Adenosquamous carcinoma | 1 | 1 | 15 | 14 |
| 8570/3 | Adenocarcinoma with squamous metaplasia | 0 | 0 | 1 | 0 |
| Total non-BAC lung adenocarcinomas | | 94 | 78 | 381 | 326 |
| 1. **Lepidic/non-mucinous BAC** | | | | | |
| 8250/3 | Bronchiolo-alveolar adenocarcinoma, NOS | 10 | 1 | 83 | 25 |
| 8252/3 | Bronchiolo-alveolar carcinoma, non-mucinous | 0 | 0 | 16 | 7 |
| Total BAC, lepidic/non-mucinous | | 10 | 1 | 99 | 32 |
| 1. **Mucinous BAC** | | | | | |
| 8253/3 | Bronchiolo-alveolar carcinoma, mucinous | 1 | 0 | 6 | 4 |
| 8254/3 | Bronchiolo-alveolar carcinoma, mixed mucinous and non-mucinous | 0 | 0 | 4 | 0 |
| Total BAC, mucinous | | 1 | 0 | 10 | 4 |
| 1. **Lung squamous cell carcinomas** | | | | | |
| 8052/3 | Papillary squamous cell carcinoma | 0 | 0 | 0 | 1 |
| 8070/3 | Squamous cell carcinoma, NOS | 24 | 33 | 214 | 176 |
| 8071/3 | Squamous cell carcinoma, keratinizing, NOS | 3 | 7 | 12 | 12 |
| 8072/3 | Squamous cell carcinoma, large cell, non-keratinizing, NOS | 4 | 2 | 3 | 2 |
| 8074/3 | Squamous cell carcinoma, spindle cell | 0 | 1 | 0 | 0 |
| 8075/3 | Squamous cell carcinoma, adenoid | 0 | 0 | 1 | 0 |
| 8076/3 | Squamous cell carcinoma, microinvasive | 0 | 1 | 0 | 0 |
| 8078/3 | Squamous cell carcinoma with horn formation | 1 | 1 | 0 | 0 |
| 8083/3 | Basaloid squamous cell carcinoma | 4 | 2 | 2 | 0 |
| 8084/3 | Squamous cell carcinoma, clear cell type | 0 | 0 | 1 | 0 |
| Total lung squamous Cell Carcinomas | | 36 | 47 | 233 | 191 |
| 1. **Other non-small cell lung carcinomas** | | | | | |
| 8010/3 | Carcinoma, NOS | 2 | 5 | 18 | 17 |
| 8012/3 | Large cell carcinoma, NOS | 1 | 0 | 22 | 30 |
| 8013/3 | Large cell neuroendocrine carcinoma | 1 | 2 | 18 | 14 |
| 8020/3 | Carcinoma, undifferentiated type, NOS | 1 | 0 | 0 | 0 |
| 8021/3 | Carcinoma, anaplastic type, NOS | 0 | 0 | 0 | 1 |
| 8022/3 | Pleomorphic carcinoma | 1 | 0 | 0 | 1 |
| 8032/3 | Spindle cell carcinoma, NOS | 0 | 0 | 1 | 1 |
| 8033/3 | Pseudosarcomatous carcinoma | 0 | 0 | 0 | 3 |
| 8046/3 | Non-small cell carcinoma | 8 | 9 | 83 | 107 |
| 8050/3 | Papillary carcinoma, NOS | 0 | 0 | 0 | 0 |
| 8980/3 | Carcinosarcoma, NOS | 0 | 0 | 1 | 1 |
| Total other non-small cell carcinomas | | 14 | 16 | 143 | 175 |
| 1. **Small cell lung carcinomas** | | | | | |
| 8041/3 | Small cell carcinoma, NOS | 21 | 35 | 115 | 130 |
| 8042/3 | Oat cell carcinoma | 2 | 2 | 8 | 3 |
| 8044/3 | Small cell carcinoma, intermediate cell | 1 | 0 | 0 | 1 |
| 8045/3 | Combined small cell carcinoma | 3 | 1 | 5 | 3 |
| Total small cell carcinomas | | 27 | 38 | 128 | 137 |
| 1. **Other neuro-endocrine tumors** | | | | | |
| 8240/3 | Carcinoid tumor, NOS | 3 | 1 | 5 | 1 |
| 8249/3 | Atypical carcinoid tumor | 1 | 2 | 7 | 10 |
| 8246/3 | Neuroendocrine carcinoma, NOS | 0 | 1 | 1 | 1 |
| Total other neuro-endocrine tumors | | 4 | 4 | 13 | 12 |
| 1. **Unclassified** **tumors** | | | | | |
| 8001/3 | Tumor cells, malignant | 0 | 0 | 0 | 1 |
| 8000/3 | Neoplasm, malignant | 4 | 6 | 22 | 15 |
| Total unclassified tumors | | 4 | 6 | 22 | 16 |

**Abbreviations:** ITALUNG: Italian Lung Study; LUSI: Lung Cancer Screening Intervention study; NLST: National Lung Screening Trial; NOS: not otherwise specified; ICD-O-3: International Classification of Diseases for Oncology, Third Edition; BAC: bronchiolo-alveolar carcinoma.

**Supplementary Table S2 Characteristics of female and male participants in ITALUNG and LUSI.**

| **Characteristic** | **ITALUNG** | | | | **LUSI** | | | |
| --- | --- | --- | --- | --- | --- | --- | --- | --- |
|  | **Control (*n* = 1,593)** | | **Screening (*n* = 1,613)^a^** | | **Control (*n* = 2,023)** | | **Screening (*n =* 2,029)** | |
|  | **Females** | **Males** | **Females** | **Males** | **Females** | **Males** | **Females** | **Males** |
|  | 554 (34.8%) | 1,039 (65.2%) | 578 (35.8%) | 1,035 (64.2%) | 716 (35.4%) | 1,307 (64.6%) | 714 (35.2%) | 1,315 (64.8%) |
| **Age, years (at randomization)** | | | | | | | | |
| Median [IQR] | 60.0  [57.0, 64.0] | 61.0  [58.0, 65.0] | 59.0  [57.0, 64.0] | 61.0  [57.0, 65.0] | 56.3  [53.0, 61.1] | 57.3  [52.9, 62.1] | 56.6  [52.7, 61.0] | 57.1  [53.1, 62.3] |
| **Smoking history (at baseline)** | | | | | | | | |
| **Current** | 406 (73.3%) | 612 (58.9%) | 432 (74.7%) | 627 (60.6%) | 474 (66.2%) | 762 (58.3%) | 476 (66.7%) | 773 (58.8%) |
| **Former** | 148 (26.7%) | 427 (41.1%) | 146 (25.3%) | 408 (39.4%) | 242 (33.8%) | 545 (41.7%) | 238 (33.3%) | 542 (41.2%) |
| **Duration, years (at baseline)** | | | | | | | | |
| Median [IQR] | 40.0  [36.0, 43.0] | 42.0  [38.0, 46.0] | 40.0  [36.0, 44.0] | 42.0  [39.0, 46.0] | 33.0  [33.0, 38.0] | 38.0  [33.0, 38.0] | 33.0  [33.0, 38.0] | 38.0  [33.0, 38.0] |
| **Time since quitting, years (at baseline)** ^b^ | | | | | | | | |
| Median [IQR] | 4.00  [2.0, 7.0] | 5.00  [3.0, 8.0] | 4.00  [2.0, 7.0] | 5.00  [3.0, 8.0] | 4.00  [1.5, 7.00] | 4.00  [1.5, 7.0] | 4.00  [1.5, 7.0] | 4.00  [1.7, 7.0] |
| **Pack years**^c^ | | | | | | | | |
| Median [IQR] | 34.5  [26.4, 43.0] | 40.0  [30.8, 50.0] | 34.0  [26.5, 43.0] | 42.0  [33.8, 54.5] | 34.2  [25.2, 43.7] | 38.7  [29.7, 53.2] | 34.2  [25.2, 43.7] | 38.7  [29.7, 53.2] |
| **Follow-up time since randomization, years** | | | | | | | | |
| Median [min, max] | 11  [0.63, 11] | 11  [0.25, 11] | 11  [0.016, 11] | 11  [0.76, 11] | 12  [1.69, 12] | 12  [0.39, 12] | 12  [1.35, 12] | 12  [0.52, 12] |
| **Follow-up time since last screening,** **years** | | | | | | | | |
| Median [min] | NA | NA | 8  [0.016] | 8  [0.76] | NA | NA | 8  [1.35] | 8  [0.52] |
| **Total person years** ^d^ | 5,867 | 10,595 | 6,149 | 10,711 | 8,568 | 15,063 | 8,582 | 15,183 |
| **Lung cancer cases, *n* (%)** | | | | | | | | |
| No lung cancer | 530 (95.7%) | 969 (93.3%) | 561 (97.1%) | 965 (93.2%) | 683 (95.4 %) | 1,244 (95.2%) | 683 (95.7%) | 1,243 (94.5%) |
| Lung cancer diagnosis ^e^ | 24 (4.3 %) | 70 (6.7 %) | 17 (2.9 %) | 70 (6.8 %) | 33 (4.6 %) | 63 (4.8%) | 31 (4.3%) | 72 (5.5%) |
| Lung cancer diagnosis ^f^ | 16 (2.9 %) | 43 (4.1 %) | 9 (1.6 %) | 46 (4.4 %) | 19 (2.7 %) | 43 (3.3%) | 26 (3.6%) | 54 (4.1%) |
| **Vital status, *n* (%)** | | | | | | | | |
| Alive | 504 (91.0%) | 864 (83.2%) | 531 (91.9%) | 893 (86.3%) | 648 (90.5%) | 1,097 (83.9%) | 665 (93.1%) | 1,096 (83.3%) |
| Deceased | 50 (9.0%) | 175 (16.8%) | 47 (8.1%) | 142 (13.7%) | 58 (8.1%) | 200 (15.3%) | 46 (6.4%) | 210 (16.0%) |
| Lung cancer ^g^ | 16 (2.9 %) | 50 (4.8 %) | 10 (1.7 %) | 43 (4.1 %) | 18 (2.5%) | 43 (3.3%) | 8 (1.1%) | 36 (2.8%) |
| Lung cancer ^h^ | 13 (2.3%) | 35 (3.4%) | 5 (0.9%) | 28 (2.7 %) | 12 (1.7%) | 27 (2.1%) | 6 (0.8%) | 24 (2.7%) |
| Other causes | 34 (6.1%) | 125 (12.0%) | 37 (6.4%) | 99 (9.6%) | 40 (5.6%) | 157 (12.0%) | 38 (5.3%) | 174 (13.2%) |
| Unknown | 0 (0.0%) | 0 (0.0%) | 0 (0.0%) | 0 (0.0%) | 10 (1.4%) | 10 (0.8%) | 3 (0.4%) | 9 (0.7%) |
| ^a^ Screening arm includes 207 individuals (“drop-outs”) who after randomization refused to undergo CT screening  ^b^ Only in former smokers  ^c^ Pack years were calculated as follows: (cigarettes per day ÷ 20) × (years smoking).  ^d^ Total person years were calculated by adding the time in years that a participant was followed since randomization until the established time limits in the study (i.e., 11 years for ITALUNG and 12 years for LUSI after randomization)  ^e^ Lung cancer cases diagnosed within 8 years after the last scheduled screening (i.e., 11 years for ITALUNG and 12 years for LUSI after randomization)  ^f^ Lung Cancer cases diagnosed within 4 years after the last scheduled screening (i.e., 7 years for ITALUNG and 8 years for LUSI after randomization)  ^g^ Mortality within 8 years after last scheduled screening, for LC cases diagnosed within 8 years after the last scheduled screening (i.e., 11 years for ITALUNG and 12 years for LUSI after randomization).  ^h^ Mortality within 6 years after last scheduled screening (i.e., 9 years for ITALUNG and 10 years for LUSI after randomization), for LC cases diagnosed within 4 years after the last scheduled screening (i.e., 7 years for ITALUNG and 8 years for LUSI after randomization).  Abbreviations: ITALUNG: Italian Lung Study; LUSI: Lung Cancer Screening Intervention study; IQR: interquartile range; min: minimum value; max: maximum value; NA: not applicable; CT: computed tomography; LC: lung cancer. | | | | | | | | |

**Supplementary Table S3. Characteristics of female and male participants in the NLST.**

| **Characteristic** | **NLST** | | | |
| --- | --- | --- | --- | --- |
|  | **Control (X-ray)**  **(*n* = 26,730)** | | **Screening**  **(*n* = 26,722)** | |
|  | **Females** | **Males** | **Females** | **Males** |
|  | 10,969 (41.0%) | 15,761 (59.0%) | 10,953 (41.0%) | 15,769 (59.0%) |
| **Age, years (at randomization).** | | | | |
| Median [IQR] | 60.0 [57.0, 64.0] | 61.0 [57.0, 65.0] | 60.0 [57.0, 64.0] | 61.0 [57.0, 65.0] |
| **Smoking history (at baseline)** | | | | |
| **Current** | 5,470 (49.9%) | 7,430 (47.1%) | 5,519 (50.4%) | 7,341 (46.6%) |
| **Former** | 5,499 (50.1%) | 8,331 (52.9%) | 5,434 (49.6%) | 8,428 (53.4%) |
| **Age started smoking, years.** |  |  |  |  |
| Median [IQR] | 17.0 [15.0, 19.0] | 16.0 [14.0, 18.0] | 17.0 [15.0, 19.0] | 16.0 [14.0, 18.0] |
| **Duration, years (at baseline).** |  |  |  |  |
| Median [IQR] | 40.0 [35.0, 44.0] | 40.0 [35.0, 45.0] | 40.0 [35.0, 44.0] | 40.0 [35.0, 45.0] |
| **Time since quitting, years**  ^a^ |  |  |  |  |
| Median [IQR] | 7.00 [3.0, 11.0] | 7.00 [3.0, 12.0] | 7.00 [3.0, 11.0] | 7.00 [3.0, 12.0] |
| **Pack years** ^b^ |  |  |  |  |
| Median [IQR] | 45.0 [38.0, 60.0] | 51.3 [40.0, 71.7] | 44.0 [38.0, 60.0] | 52.0 [41.0, 72.0] |
| **Follow-up time since randomization, years** | |  |  |  |
| Median [IQR] | 6.65 [6.2, 6.9] | 6.64 [0, 8.13] | 6.66 [0, 7.86] | 6.65 [0, 8.17] |
| **Total person years**  ^c^ | 70,529.6 | 99,941.8 | 70,949.1 | 100,580.4 |
| **Lung cancer cases** | | | | |
| No lung cancer | 10,574 (96.4%) | 15,187 (96.4%) | 10,519 (96.0%) | 15,114 (95.8%) |
| Lung cancer diagnosis | 395 (3.6%) | 574 (3.6%) | 434 (4.0%) | 655 (4.2%) |
| Lung cancer diagnosis ^d^ | 368 (3.4%) | 525 (3.3%) | 408 (3.7%) | 621 (3.9%) |
| **Vital status** | | | | |
| Alive *n* (%) ^c^ | 9,174 (83.6%) | 12,136 (77.0%) | 9,219 (84.2%) | 12,207 (77.4%) |
| Deceased | 1,795 (16.4%) | 3,625 (23.0%) | 1,734 (15.8%) | 3,562 (22.6%) |
| -Lung cancer deaths, *n* (%) ^e^ | 242 (2.2%) | 379 (2.4%) | 194 (1.8%) | 360 (2.3%) |
| -Lung cancer deaths, *n* (%) ^f^ | 217 (2.0%) | 344 (2.2%) | 169 (1.5%) | 328 (2.1%) |
| -Other causes, *n* | 1,553 (14.1%) | 3,264 (20.7%) | 1,540 (14.1%) | 3,202 (20.3%) |
| ^a^ Only in former smokers  ^b^ Pack years were calculated as follows: (cigarettes per day ÷ 20) × (years smoking).  ^c^ Total person years were calculated by adding the time in years that a participant was followed since randomization until the date the participant was last known to be alive (as defined in the data dictionary from the NLST)  ^d^ Restricted to those from Lung Cancer cases diagnosed within 4 years after the last scheduled screening (i.e., 6 years after randomization)  ^e^ Up to 2015, this information does not reflect updated death data for all centers: one study center with fewer than 1,200 participants did not submit participants for the updated NDI search  ^f^ Restricted to those from Lung Cancer cases diagnosed within 4 years after the last scheduled screening (i.e., 6 years after randomization), and Lung Cancer mortality within 6 years after the last scheduled screening round (i.e., 8 years after randomization).  Abbreviations: NLST: National Lung Screening Trial; IQR: interquartile range; NDI: National Death Index. | | | | |

**Supplementary Table S4. Incident lung cancer cases (I; *n* = 380) and lung cancer deaths (D; *n* = 224) in the combined screening and control arms of ITALUNG-LUSI, by histology and stage at diagnosis, up to 8 years after last scheduled screening participation.**

| ***Histology and stage*** | **Males** | | | | | | | **Females** | | | | | | | | |
| --- | --- | --- | --- | --- | --- | --- | --- | --- | --- | --- | --- | --- | --- | --- | --- | --- |
|  | **Screening arm** | | | | **Control arm** | | **Difference** ^a^ | **Screening arm** | | | | | **Control arm** | | | **Difference** ^a^ |
|  | **Positive screening test** | | **No positive screening test** | |  |  |  | **Positive screening test** | | | **No positive screening test** | |  |  |  |  |
|  | **I** | **D** | **I** | **D** | **I** | **D** |  | **I** | **D** | | **I** | **D** | **I** | | **D** |  |
| ***non-BAC LUAD*** ^b^ | | | | | | | | | | | | | | | | |
| Stage I | 29 | 7 | 5 | 1 | 10 | 2 | +24 /+6 | 17 | 3 | | 2 | 0 | 3 | | 0 | +16 / +3 |
| Stage II | 5 | 3 | 1 | 0 | 4 | 1 | +2 / +2 | 0 | 0 | | 1 | 0 | 6 | | 1 | -5 / -1 |
| Stage III | 5 | 4 | 3 | 2 | 8 | 5 | 0 / +1 | 0 | 0 | | 2 | 0 | 6 | | 3 | -4 / -3 |
| Stage IV | 3 | 3 | 12 | 9 | 26 | 24 | -11/ -12 | 1 | 1 | | 4 | 3 | 7 | | 6 | -2 / -2 |
| Non-specified | 0 | 0 | 4 | 1 | 6 | 2 | -2 / -1 | 0 | 0 | | 0 | 0 | 2 | | 2 | -2 / -2 |
| All stages | 42 (57.5%) | NA | 25 (36.2%) | NA | 54 (40.6%) | NA | +13 / -4 | 18 (64.3%) | | NA | 9 (45.0%) | NA | 24 (42.1%) | NA | | +3 / -5 |
| ***Lepidic/non-mucinous BAC*** ^c^ | | | | | | | | | | | | | | | | |
| Stage I | 4 | 1 | 0 | 0 | 0 | 0 | +4 / +1 | 4 | 0 | | 0 | 0 | 0 | | 0 | +4 / 0 |
| Stage II | 0 | 0 | 0 | 0 | 0 | 0 | 0 / 0 | 1 | 0 | | 0 | 0 | 0 | | 0 | +1 / 0 |
| Stage III | 0 | 0 | 0 | 0 | 0 | 0 | 0 / 0 | 0 | 0 | | 0 | 0 | 0 | | 0 | 0 / 0 |
| Stage IV | 0 | 0 | 0 | 0 | 0 | 0 | 0 / 0 | 0 | 0 | | 1 | 1 | 1 | | 1 | 0 / 0 |
| Non-specified | 0 | 0 | 0 | 0 | 0 | 0 | 0 / 0 | 0 | 0 | | 0 | 0 | 0 | | 0 | 0 / 0 |
| All stages | 4 (5.5%) | NA | 0 (0.0%) | NA | 0 (0.0%) | NA | +4 / +1 | 5 (17.9%) | NA | | 1 (5.0%) | NA | 1 (1.8%) | | NA | +5 / 0 |
| ***BAC mucinous*** ^d^ | | | | | | | | | | | | | | | | |
| Stage I | 0 | 0 | 0 | 0 | 0 | 0 | 0 / 0 | 0 | 0 | | 0 | 0 | 0 | | 0 | 0 / 0 |
| Stage II | 0 | 0 | 0 | 0 | 0 | 0 | 0 / 0 | 0 | 0 | | 0 | 0 | 0 | | 0 | 0 / 0 |
| Stage III | 0 | 0 | 0 | 0 | 0 | 0 | 0 / 0 | 0 | 0 | | 0 | 0 | 0 | | 0 | 0 / 0 |
| Stage IV | 0 | 0 | 1 | 0 | 0 | 0 | +1 / 0 | 0 | 0 | | 0 | 0 | 0 | | 0 | 0 / 0 |
| Non-specified | 0 | 0 | 0 | 0 | 0 | 0 | 0 / 0 | 0 | 0 | | 0 | 0 | 0 | | 0 | 0 / 0 |
| All stages | 0 (0.0%) | NA | 1 (1.4%) | NA | 0 (0.0%) | NA | +1 / 0 | 0 (0.0%) | NA | | 0 (0.0%) | NA | 0 (0.0%) | | NA | 0 / 0 |
| ***LUSC*** ^e^ | | | | | | | | | | | | | | | | |
| Stage I | 8 | 2 | 4 | 2 | 4 | 1 | +8 / +3 | 0 | 0 | | 0 | 0 | 1 | | 0 | -1 / 0 |
| Stage II | 2 | 1 | 0 | 0 | 5 | 0 | -3 / +1 | 1 | 0 | | 0 | 0 | 2 | | 0 | -1 / 0 |
| Stage III | 0 | 0 | 3 | 1 | 10 | 9 | -7 / -8 | 1 | 1 | | 3 | 2 | 2 | | 1 | +2 / +2 |
| Stage IV | 5 | 5 | 5 | 4 | 10 | 9 | 0 / 0 | 0 | 0 | | 2 | 2 | 4 | | 2 | -2 / 0 |
| Non-specified | 0 | 0 | 2 | 1 | 7 | 7 | -5 / -6 | 0 | 0 | | 0 | 0 | 2 | | 1 | -2 / -1 |
| All stages | 15 (20.5%) | NA | 14 (20.3%) | NA | 36 (27.1%) | NA | -7 / -10 | 2 (7.1%) | NA | | 5 (25%) | NA | 11 (19.3%) | | NA | -4 / +1 |
| ***Other NSCLC*** ^f^ | | | | | | | | | | | | | | | | |
| Stage I | 1 | 0 | 0 | 0 | 1 | 0 | 0 / 0 | 1 | 1 | | 1 | 0 | 0 | | 0 | +2 / +1 |
| Stage II | 1 | 1 | 0 | 0 | 0 | 0 | +1 / +1 | 0 | 0 | | 0 | 0 | 0 | | 0 | 0 / 0 |
| Stage III | 1 | 0 | 1 | 1 | 2 | 0 | 0 / +1 | 0 | 0 | | 0 | 0 | 1 | | 1 | -1 / -1 |
| Stage IV | 0 | 0 | 5 | 5 | 6 | 6 | -1 / -1 | 0 | 0 | | 1 | 1 | 4 | | 3 | -3 / -2 |
| Non-specified | 0 | 0 | 2 | 1 | 2 | 2 | 0 / -1 | 0 | 0 | | 0 | 0 | 0 | | 0 | 0 / 0 |
| All stages | 3 (4.1%) | NA | 8 (11.6%) | NA | 11 (8.3%) | NA | 0 / 0 | 1 (3.6%) | NA | | 2 (10.0%) | NA | 5 (8.8%) | | NA | -2 / -2 |
| ***SCLC*** ^g^ | | | | | | | | | | | | | | | | |
| Stage I | 1 | 0 | 0 | 0 | 0 | 0 | +1 / 0 | 0 | 0 | | 0 | 0 | 2 | | 2 | -2 / -2 |
| Stage II | 0 | 0 | 0 | 0 | 1 | 1 | -1 / -1 | 0 | 0 | | 0 | 0 | 0 | | 0 | 0 / 0 |
| Stage III | 3 | 2 | 1 | 1 | 4 | 2 | 0 / +1 | 1 | 1 | | 2 | 1 | 3 | | 2 | 0 / 0 |
| Stage IV | 1 | 1 | 16 | 16 | 16 | 13 | +1 / +4 | 0 | 0 | | 1 | 1 | 7 | | 7 | -6 / -6 |
| Non-specified | 0 | 0 | 1 | 1 | 5 | 3 | -4 / -2 | 0 | 0 | | 0 | 0 | 0 | | 0 | 0 / 0 |
| All stages | 5 (6.8%) | NA | 18 (26.1%) | NA | 26 (19.5%) | NA | -3 / +2 | 1 (3.6%) | NA | | 3 (15.0%) | NA | 12 (21.1%) | | NA | -8 / -8 |
| ***Other neuroendocrine*** ^h^ | | | | | | | | | | | | | | | | |
| Stage I | 3 | 0 | 0 | 0 | 0 | 0 | +3 / 0 | 0 | 0 | | 0 | 0 | 1 | | 0 | -1 / 0 |
| Stage II | 0 | 0 | 0 | 0 | 0 | 0 | 0 / 0 | 1 | 0 | | 0 | 0 | 0 | | 0 | +1 / 0 |
| Stage III | 0 | 0 | 0 | 0 | 0 | 0 | 0 / 0 | 0 | 0 | | 0 | 0 | 1 | | 0 | -1 / 0 |
| Stage IV | 0 | 0 | 0 | 0 | 2 | 2 | -2 / -2 | 0 | 0 | | 0 | 0 | 0 | | 0 | 0 / 0 |
| Non-specified | 0 | 0 | 0 | 0 | 0 | 0 | 0 / 0 | 0 | 0 | | 0 | 0 | 0 | | 0 | 0 / 0 |
| All stages | 3 (4.1%) | NA | 0 (0.0%) | NA | 2 (1.5%) | NA | +1 / -2 | 1 (3.6%) | NA | | 0 (0,0%) | NA | 2 (3.5%) | | NA | -1 / 0 |
| ***Unclassified*** ^i^ | | | | | | | | | | | | | | | | |
| Stage I | 0 | 0 | 0 | 0 | 0 | 0 | 0 / 0 | 0 | 0 | | 0 | 0 | 0 | | 0 | 0 / 0 |
| Stage II | 0 | 0 | 0 | 0 | 0 | 0 | 0 / 0 | 0 | 0 | | 0 | 0 | 0 | | 0 | 0 / 0 |
| Stage III | 0 | 0 | 0 | 0 | 1 | 1 | -1 / -1 | 0 | 0 | | 0 | 0 | 0 | | 0 | 0 / 0 |
| Stage IV | 0 | 0 | 2 | 2 | 2 | 2 | 0 / 0 | 0 | 0 | | 0 | 0 | 2 | | 2 | -2 / -2 |
| Non-specified | 1 | 1 | 1 | 0 | 1 | 1 | +1 / 0 | 0 | 0 | | 0 | 0 | 0 | | 0 | 0 / 0 |
| All stages | 1 (1.4%) | NA | 3 (4.3%) |  | 4 (3.0%) | NA | 0 / -1 | 0 (0%) | NA | | 0 (0%) | NA | 2 (3.5%) | | NA | -2 / -2 |
| ***All histologies combined*** | | | | | | | | | | | | | | | | |
| **Total** | 73 | 31 | 69 | 48 | 133 | 93 | +9 / -14 | 28 | 7 | | 20 | 11 | 57 | | 34 | -9 / -16 |
| ^a^ Difference between the incident lung cancer cases (left side) and mortality cases (right side) in the screening arm (positive screening test + non-positive screening test) in comparison to the control arm.  ^b^ Adenocarcinomas without BAC: ICD-O-3 = 8140, 8230, 8255, 8260, 8310, 8480, 8490, 8550, 8560.  ^c^ Lepidic/non-mucinous bronchiolo-alveolar carcinoma: ICD-O-3 = 8250, 8252.  ^d^ Mucinous bronchiolo-alveolar carcinoma: ICD-O-3 = 8253, 8254.  ^e^ Squamous cell carcinoma: ICD-O-3 = 8052, 8070, 8071, 8072, 8074, 8076, 8078, 8083.  ^f^ Other non-small cell carcinoma: ICD-O-3 = 8010,8012, 8013, 8020, 8021, 8022, 8032, 8033, 8046, 8050, 8980.  ^g^ Small cell carcinoma: ICD-O-3 = 8041, 8042, 8044, 8045.  ^h^ Other neuroendocrine tumors: ICD-O-3 = 8240, 8246, 8249.  ^i^ Unclassified tumors: ICD-O-3 = 8000, 8001.  Abbreviations: ITALUNG: Italian Lung Study; LUSI: Lung Cancer Screening Intervention study; BAC: bronchiolo-alveolar carcinoma; ICD-O-3: International Classification of Diseases for Oncology, Third Edition; LUAD: lung adenocarcinoma; LUSC: lung squamous cell carcinomas; NSCLC: non-small cell lung cancers; SCLC: small-cell lung cancer; NA: not applicable. | | | | | | | | | | | | | | | | |

**Supplementary Table S5. Cases of incident lung cancer (I; total *n =* 1,922) diagnosed up to 4 years after scheduled final screening, and corresponding lung cancer deaths with 6 years after final screening (D; total *n =* 1,058) among female and male participants in the LDCT and CXR arms of the NLST, by histology and stage at diagnosis.**

| ***Histology and stage*** | **Males** | | | | | | | **Females** | | | | | | | | |
| --- | --- | --- | --- | --- | --- | --- | --- | --- | --- | --- | --- | --- | --- | --- | --- | --- |
|  | **Screening arm** | | | | **Control arm** | | **Difference** ^a^ | **Screening arm** | | | | | **Control arm** | | | **Difference** ^a^ |
|  | **Positive screening test** | | **No positive screening test** | |  |  |  | **Positive screening test** | | | **No positive screening test** | |  |  |  |  |
|  | **I** | **D** | **I** | **D** | **I** | **D** |  | **I** | **D** | | **I** | **D** | **I** | | **D** |  |
| ***non-BAC LUAD*** ^b^ | | | | | | | | | | | | | | | | |
| Stage I | 95 | 16 | 27 | 11 | 73 | 26 | +49 /+1 | 89 | 15 | | 15 | 1 | 53 | | 12 | +51 / +4 |
| Stage II | 16 | 8 | 1 | 0 | 13 | 6 | + 4 / +2 | 8 | 2 | | 2 | 1 | 12 | | 5 | -2 / -2 |
| Stage III | 21 | 13 | 13 | 11 | 37 | 26 | - 3 / -2 | 15 | 7 | | 12 | 9 | 29 | | 16 | -2 / -0 |
| Stage IV | 17 | 16 | 25 | 23 | 66 | 64 | -24 / -25 | 6 | 6 | | 17 | 14 | 43 | | 41 | -20 / -21 |
| Non-specified | 0 | 0 | 0 | 0 | 0 | 0 | 0 / 0 | 2 | 1 | | 0 | 0 | 0 | | 0 | +2 / +1 |
| All stages | 149 (38.8%) | NA | 66 (27.8%) | NA | 189 (36.0%) | NA | +26 / -24 | 120 (45.3%) | | NA | 46 (32.2%) | NA | 137 (37.2%) | NA | | +29/-18 |
| ***Lepidic/non-mucinous BAC*** ^c^ | | | | | | | | | | | | | | | | |
| Stage I | 26 | 3 | 7 | 3 | 7 | 1 | +26 / +5 | 43 | 0 | | 5 | 1 | 9 | | 2 | +39 / -1 |
| Stage II | 3 | 1 | 0 | 0 | 1 | 0 | +2 / +1 | 1 | 0 | | 0 | 0 | 2 | | 1 | -1 / -1 |
| Stage III | 3 | 2 | 0 | 0 | 4 | 4 | -1 / -2 | 5 | 2 | | 1 | 0 | 4 | | 1 | +2 / +1 |
| Stage IV | 1 | 1 | 1 | 1 | 4 | 3 | -2 / -1 | 3 | 1 | | 0 | 0 | 1 | | 0 | +2 / +1 |
| Non-specified | 0 | 0 | 0 | 0 | 0 | 0 | 0 / 0 | 0 | 0 | | 0 | 0 | 0 | | 0 | 0 / 0 |
| All stages | 33 (8.6%) | NA | 8 (3.4%) | NA | 16 (3.0%) | NA | +25 / +3 | 52 (19.6%) | NA | | 6 (4.2%) | NA | 16 (4.3%) | | NA | +42 / 0 |
| ***Mucinous BAC*** ^d^ | | | | | | | | | | | | | | | | |
| Stage I | 3 | 1 | 0 | 0 | 2 | 0 | +1 / +1 | 5 | 1 | | 0 | 0 | 1 | | 0 | +4 / +1 |
| Stage II | 0 | 0 | 0 | 0 | 0 | 0 | 0 / 0 | 0 | 0 | | 0 | 0 | 0 | | 0 | 0 / 0 |
| Stage III | 0 | 0 | 0 | 0 | 0 | 0 | 0 / 0 | 0 | 0 | | 0 | 0 | 0 | | 0 | 0 / 0 |
| Stage IV | 2 | 1 | 0 | 0 | 0 | 0 | +2 / +1 | 0 | 0 | | 0 | 0 | 1 | | 0 | -1 / 0 |
| Non-specified | 0 | 0 | 0 | 0 | 0 | 0 | 0 / 0 | 0 | 0 | | 0 | 0 | 0 | | 0 | 0 / 0 |
| All stages | 5 (1.3%) | NA | 0 (0.0%) | NA | 2 (0.0%) | NA | +3 / +2 | 5 (1.9%) | NA | | 0 (0.0%) | NA | 2 (0.5%) | | NA | +3 / +1 |
| ***LUSC*** ^e^ | | | | | | | | | | | | | | | | |
| Stage I | 71 | 16 | 26 | 4 | 44 | 9 | +53/+11 | 15 | 3 | | 11 | 2 | 29 | | 3 | -3 / +2 |
| Stage II | 10 | 4 | 10 | 6 | 19 | 7 | +1 / +3 | 0 | 0 | | 4 | 1 | 4 | | 1 | 0 / 0 |
| Stage III | 17 | 15 | 21 | 19 | 32 | 21 | +6 / +13 | 12 | 10 | | 7 | 4 | 16 | | 9 | +3 / +5 |
| Stage IV | 8 | 7 | 10 | 9 | 32 | 27 | -14/ -11 | 1 | 1 | | 8 | 8 | 14 | | 11 | -5 / -2 |
| Non-specified | 2 | 2 | 0 | 0 | 1 | 0 | +1 / +2 | 0 | 0 | | 0 | 0 | 0 | | 0 | 0 / 0 |
| All stages | 108 (28.1%) | NA | 67 (28.3%) | NA | 128 (24.4%) | NA | +47/ +18 | 28 (10.6%) | NA | | 30 (21.0%) | NA | 63 (17.1%) | | NA | -5 / +5 |
| ***Other NSCLC*** ^f^ | | | | | | | | | | | | | | | | |
| Stage I | 25 | 4 | 2 | 2 | 17 | 4 | +10 / +2 | 23 | 7 | | 6 | 2 | 15 | | 4 | +14 / +5 |
| Stage II | 3 | 2 | 3 | 1 | 4 | 3 | +2 / 0 | 0 | 0 | | 1 | 0 | 4 | | 2 | -3 / -2 |
| Stage III | 12 | 7 | 15 | 13 | 28 | 19 | -1 / +1 | 5 | 5 | | 8 | 5 | 26 | | 19 | -13 / -9 |
| Stage IV | 12 | 11 | 12 | 11 | 53 | 49 | -29 / -27 | 8 | 8 | | 6 | 6 | 26 | | 24 | -12 / -10 |
| Non-specified | 1 | 1 | 0 | 0 | 0 | 0 | +1 / +1 | 0 | 0 | | 1 | 1 | 2 | | 1 | -1 / 0 |
| All stages | 53 (13.8%) | NA | 32 (13.5%) | NA | 102 (19.4%) | NA | -17 / -23 | 36 (13.6%) | NA | | 22 (15.4%) | NA | 73 (19.8%) | | NA | -15 / -16 |
| ***SCLC*** ^g^ | | | | | | | | | | | | | | | | |
| Stage I | 1 | 1 | 2 | 2 | 5 | 3 | -2 / -0 | 1 | 0 | | 1 | 0 | 6 | | 4 | -4 / -4 |
| Stage II | 3 | 1 | 0 | 0 | 5 | 2 | -2 / -1 | 2 | 1 | | 0 | 0 | 2 | | 1 | 0 / 0 |
| Stage III | 12 | 11 | 14 | 11 | 19 | 17 | +7 / +5 | 5 | 5 | | 13 | 9 | 21 | | 17 | -3 / -3 |
| Stage IV | 13 | 12 | 34 | 32 | 43 | 41 | +4 / +3 | 8 | 8 | | 14 | 14 | 34 | | 33 | -12 / -11 |
| Non-specified | 2 | 1 | 0 | 0 | 1 | 1 | +1 / 0 | 1 | 0 | | 2 | 1 | 1 | | 1 | +2/ 0 |
| All stages | 31 (8.1%) | NA | 50 (21.1%) | NA | 73 (13.9%) | NA | +8 / +7 | 17 (6.4%) | NA | | 30 (21.0%) | NA | 64 (17.4%) | | NA | -17 / -18 |
| ***Other neuroendocrine*** ^h^ | | | | | | | | | | | | | | | | |
| Stage I | 0 | 0 | 3 | 1 | 3 | 1 | 0 / 0 | 0 | 0 | | 1 | 0 | 3 | | 0 | -2 / 0 |
| Stage II | 0 | 0 | 0 | 0 | 0 | 0 | 0 / 0 | 0 | 0 | | 0 | 0 | 0 | | 0 | 0 / 0 |
| Stage III | 1 | 1 | 0 | 0 | 2 | 2 | -1 / -1 | 0 | 0 | | 0 | 0 | 1 | | 0 | -1 / 0 |
| Stage IV | 0 | 0 | 3 | 3 | 1 | 1 | +2 / +2 | 0 | 0 | | 0 | 0 | 2 | | 2 | -2 / -2 |
| Non-specified | 2 | 0 | 0 | 0 | 0 | 0 | +2 / 0 | 3 | 0 | | 0 | 0 | 0 | | 0 | +3 / 0 |
| All stages | 3 (0.8%) | NA | 6 (2.5%) | NA | 6 (1.1%) | NA | +3 / +1 | 3 (1.1%) | NA | | 1 (0.7%) | NA | 6 (1.6%) | | NA | -2 / -2 |
| ***Unclassified*** ^i^ | | | | | | | | | | | | | | | | |
| Stage I | 0 | 0 | 2 | 0 | 5 | 3 | -3 / -3 | 3 | 2 | | 2 | 1 | 2 | | 2 | +3/ +1 |
| Stage II | 0 | 0 | 0 | 0 | 0 | 0 | 0 / 0 | 0 | 0 | | 0 | 0 | 0 | | 0 | 0 / 0 |
| Stage III | 0 | 0 | 1 | 1 | 2 | 2 | -1 / -1 | 1 | 1 | | 1 | 0 | 1 | | 1 | +1 / 0 |
| Stage IV | 1 | 1 | 5 | 4 | 1 | 1 | +5 / +4 | 0 | 0 | | 5 | 3 | 4 | | 4 | +1 / -1 |
| Non-specified | 1 | 1 | 0 | 0 | 1 | 1 | 0 / 0 | 0 | 0 | | 0 | 0 | 0 | | 0 | 0 / 0 |
| All stages | 2 (0.5%) | NA | 8 (3.4%) | NA | 9 (1.7%) | NA | +1 / 0 | 4 (1.5%) | NA | | 8 (5.6%) | NA | 7 (1.9%) | | NA | +5 / 0 |
| ***All histologies combined*** | | | | | | | | | | | | | | | | |
| **Total** | 384 | 160 | 237 | 168 | 525 | 344 | +96 / -16 | 265 | 86 | | 143 | 83 | 368 | | 217 | +40 / -48 |
| ^a^ Difference between the incident lung cancer cases (left side) and mortality cases (right side) in the screening arm (positive screening test + non-positive screening test) in comparison to the control arm.  ^b^ Adenocarcinomas without BAC: ICD-O-3 = 8140, 8230, 8255, 8260, 8310, 8480, 8490, 8550, 8560.  ^c^ Lepidic/non-mucinous bronchiolo-alveolar carcinoma: ICD-O-3 = 8250, 8252.  ^d^ Mucinous bronchiolo-alveolar carcinoma: ICD-O-3 = 8253, 8254.  ^e^ Squamous cell carcinoma: ICD-O-3 = 8052, 8070, 8071, 8072, 8074, 8076, 8078, 8083.  ^f^ Other non-small cell carcinoma: ICD-O-3 = 8010,8012, 8013, 8020, 8021, 8022, 8032, 8033, 8046, 8050, 8980.  ^g^ Small cell carcinoma: ICD-O-3 = 8041, 8042, 8044, 8045.  ^h^ Other neuroendocrine tumors: ICD-O-3 = 8240, 8246, 8249.  ^i^ Unclassified tumors: ICD-O-3 = 8000, 8001.  Abbreviations: NLST: National Lung Screening Trial; LDCT: low-dose computed tomography; CXR: control arm; BAC: bronchiolo-alveolar carcinoma; ICD-O-3: International Classification of Diseases for Oncology, Third Edition; LUAD: lung adenocarcinoma; LUSC: lung squamous cell carcinomas; NSCLC: non-small cell lung cancers; SCLC: small-cell lung cancer; NA: not applicable. | | | | | | | | | | | | | | | | |

**Supplementary Table 6. Cumulative lung cancer mortality and screening-related hazard ratios for participants with ≥ 40 vs. < 40 pack years, for deaths with corresponding LC diagnosis within maximally 6 years after scheduled final screening (total *n* = 1,046), by sex, histology and follow-up time after final screening; ITALUNG-LUSI and NLST combined.**

| **Years of follow-up for mortality, since the final scheduled screening** | **Males + Females** | | | | **Males** | | | | **Females** | | | |
| --- | --- | --- | --- | --- | --- | --- | --- | --- | --- | --- | --- | --- |
|  | **Screening**  **(*n* = 21,488)** | **Control**  **(*n* = 21,371)** | **Δ** | **HR**  **(95% CI)^a^** | **Screening**  **(*n* = 13,635)** | **Control**  **(*n* = 13,435)** | **Δ** | **HR**  **(95% CI) ^a^** | **Screening**  **(*n* = 7,853)** | **Control**  **(*n =* 7,936)** | **Δ** | **HR**  **(95% CI) ^a^** |
| **≥40 pack-years** | | | | | | | | | | | | |
| ***All sub-types ^b^*** | | | | | | | | | | | | |
| **2** | 234 | 280 | -46 | **0.84**  **(0.70-0.99)** | 171 | 185 | -14 | 0.92  (0.75-1.13) | 63 | 95 | -32 | **0.67**  **(0.49-0.92)** |
| **4** | 385 | 478 | -93 | **0.79**  **(0.69-0.91)** | 275 | 310 | -35 | 0.86  (0.74-1.02) | 111 | 168 | -58 | **0.66**  **(0.52-0.84)** |
| **6** | 488 | 558 | -70 | **0.86**  **(0.77-0.98)** | 341 | 357 | -16 | 0.93  (0.81-1.08) | 147 | 201 | -54 | **0.74**  **(0.60-0.91)** |
| ***Non-BAC LUAD ^c^*** | | | | | | | | | | | | |
| **2** | 72 | 87 | -15 | 0.81  (0.59-1.11) | 54 | 57 | -3 | 0.91  (0.63-1.33) | 18 | 30 | -12 | 0.61  (0.34-1.09) |
| **4** | 117 | 153 | -36 | **0.75**  **(0.59-0.96)** | 78 | 100 | -22 | 0.76  (0.57-1.03) | 39 | 53 | -14 | 0.73  (0.48-1.10) |
| **6** | 155 | 183 | -28 | 0.84  (0.68-1.04) | 105 | 118 | -13 | 0.87  (0.67-1.13) | 50 | 65 | -15 | 0.77  (0.54-1.12) |
| ***LUSC ^d^*** | | | | | | | | | | | | |
| **2** | 52 | 43 | +9 | 1.23  (0.82-1.84) | 41 | 32 | +9 | 1.30  (0.81-2.07) | 11 | 11 | 0 | 1.11  (0.44-2.34) |
| **4** | 83 | 80 | +3 | 1.01  (0.75-1.38) | 69 | 62 | +7 | 1.07  (0.76-1.51) | 14 | 18 | -4 | 0.80  (0.41-1.57) |
| **6** | 102 | 96 | +6 | 1.05  (0.79-1.39) | 81 | 73 | +8 | 1.08  (0.79-1.49) | 21 | 23 | -2 | 0.92  (0.51-1.67) |
| ***Other NSCLC ^e^*** | | | | | | | | | | | | |
| **2** | 40 | 70 | -30 | **0.57**  **(0.38-0.84)** | 24 | 45 | -21 | **0.51**  **(0.31-0.84)** | 16 | 24 | -8 | 0.68  (0.36-1.27) |
| **4** | 61 | 109 | -48 | **0.55**  **(0.41-0.76)** | 39 | 70 | -31 | **0.55**  **(0.37-0.81)** | 22 | 39 | -17 | **0.57**  **(0.34-0.96)** |
| **6** | 77 | 120 | -43 | **0.64**  **(0.48-0.85)** | 48 | 75 | -27 | **0.63**  **(0.44-0.90)** | 29 | 45 | -16 | 0.65  (0.41-1.04) |
| ***SCLC ^f^*** | | | | | | | | | | | | |
| **2** | 60 | 69 | -9 | 0.90  (0.64-1.28) | 44 | 43 | +1 | 1.07 (0.71-1.63) | 16 | 26 | -10 | 0.62 (0.33-1.16) |
| **4** | 95 | 112 | -17 | 0.84  (0.64-1.10) | 67 | 65 | +2 | 1.01  (0.72-1.42) | 28 | 47 | -19 | **0.60**  **(0.38-0.96)** |
| **6** | 118 | 129 | -11 | 0.90  (0.70-1.16) | 81 | 73 | +8 | 1.08  (0.79-1.49) | 37 | 56 | -19 | 0.67  (0.44-1.00) |
| **<40 pack-years** | | | | | | | | | | | | |
| ***All sub-types ^b^*** | | | | | | | | | | | | |
| **2** | 37 | 43 | -6 | 0.82  (0.53-1.27) | 24 | 24 | 0 | 0.91  (0.51-1.62) | 13 | 19 | -6 | 0.72  (0.36-1.43) |
| **4** | 58 | 79 | -21 | 0.76  (0.54-1.06) | 34 | 42 | -8 | 0.86  (0.55-1.35) | 24 | 37 | -13 | 0.65  (0.39-1.09) |
| **6** | 72 | 90 | -18 | 0.80  (0.59-1.09) | 39 | 49 | -10 | 0.83  (0.54-1.26) | 33 | 41 | -8 | 0.78  (0.49-1.23) |
| **Non-BAC LUAD ^c^** | | | | | | | | | | | | |
| **2** | 12 | 21 | -9 | 0.55  (0.27-1.10) | 8 | 10 | -2 | 0.75  (0.30-1.87) | 4 | 11 | -7 | 0.36  (0.11-1.12) |
| **4** | 18 | 34 | -16 | **0.53**  **(0.30-0.94)** | 10 | 19 | -9 | 0.57  (0.27-1.24) | 8 | 15 | -7 | 0.49  (0.21-1.14) |
| **6** | 24 | 40 | -16 | **0.60**  **(0.36-0.99)** | 13 | 22 | -9 | 0.61  (0.31-1.22) | 11 | 18 | -7 | 0.60  (0.28-1.26) |
| ***LUSC ^d^*** | | | | | | | | | | | | |
| **2** | 13 | 6 | +7 | 2.01  (0.75-5.35) | 8 | 5 | +3 | 1.45  (0.46-4.57) | 5 | 1 | +4 | 4.92  (0.57-42.10) |
| **4** | 19 | 12 | +7 | 1.59  (0.77-3.27) | 11 | 9 | +2 | 1.27  (0.53-3.07) | 8 | 3 | +5 | 2.61  (0.69-9.84) |
| **6** | 21 | 13 | +8 | 1.62  (0.81-3.24) | 11 | 10 | +1 | 1.14  (0.49-2.69) | 10 | 3 | +7 | 3.26  (0.90-11.86) |
| ***Other NSCLC ^e^*** | | | | | | | | | | | | |
| **2** | 8 | 4 | +4 | 1.81  (0.61-5.41) | 5 | 3 | +2 | 1.73  (0.41-7.22) | 3 | 2 | +1 | 1.93  (0.35-10.52) |
| **4** | 12 | 14 | -2 | 0.86  (0.40-1.86) | 7 | 5 | +2 | 1.45  (0.46-4.56) | 5 | 9 | -4 | 0.54  (0.18-1.60) |
| **6** | 14 | 16 | -2 | 0.88  (0.43-1.80) | 8 | 7 | +1 | 1.18  (0.43-3.27) | 6 | 9 | -3 | 0.64  (0.23-1.80) |
| ***SCLC ^f^*** | | | | | | | | | | | | |
| **2** | 3 | 8 | -5 | 0.25  (0.05-1.17) | 2 | 5 | -3 | 0.21  (0.02-1.77) | 1 | 3 | -2 | 0.32  (0.03-3.06) |
| **4** | 7 | 13 | -6 | 0.54  (0.21-1.35) | 4 | 7 | -3 | 0.59  (0.17-2.02) | 3 | 6 | -3 | 0.48  (0.12-1.93) |
| **6** | 8 | 14 | -6 | 0.57  (0.24-1.36) | 5 | 7 | -2 | 0.74  (0.23-2.33) | 3 | 7 | -4 | 0.41  (0.11-1.60) |

HR was estimated using the proportional hazards model. Bold figures indicate hazard rates that significantly differ from 1.00 (*P* < 0.05).

^a^ Adjusted by age.

^b^ All subtypes: adenocarcinomas without BAC: ICD-O-3 = 8140, 8230, 8255, 8260, 8310, 8480, 8490, 8550, 8560; squamous cell carcinoma: ICD-O-3 = 8052, 8070, 8071, 8072, 8074, 8076, 8078, 8083; other non-small cell carcinoma: ICD-O-3 = 8010,8012, 8013, 8020, 8021, 8022, 8032, 8033, 8046, 8050, 8980; small cell carcinoma: ICD-O-3 = 8041, 8042, 8044, 8045; lepidic/non-mucinous bronchiolo-alveolar carcinoma (BAC): ICD-O-3 = 8250, 8252; mucinous bronchiolo-alveolar carcinoma (BAC): ICD-O-3 = 8253, 8254; other neuroendocrine: ICD-O-3 = 8240, 8246, 8249 and unclassified tumors: ICD-O-3 = 8000, 8001.

^c^ Adenocarcinomas without BAC: ICD-O-3 = 8140, 8230, 8255, 8260, 8310, 8480, 8490, 8550, 8560.

^d^ Squamous cell carcinoma: ICD-O-3 = 8052, 8070, 8071, 8072, 8074, 8076, 8078, 8083.

^e^ Other non-small cell carcinoma: ICD-O-3 = 8010,8012, 8013, 8020, 8021, 8022, 8032, 8033, 8046, 8050, 8980.

^f^ Small cell carcinoma: ICD-O-3 = 8041, 8042, 8044, 8045.

Abbreviations: LUSI: Lung Cancer Screening Intervention study; ITALUNG: Italian Lung Study; NLST: National Lung Screening Trial; HR: hazard ratio; CI: confidence interval; Δ: Difference between screening and control arm; BAC: bronchiolo-alveolar carcinoma; ICD-O-3: International Classification of Diseases for Oncology, Third edition; LUAD: lung adenocarcinoma; LUSC: lung squamous cell carcinomas; NSCLC: non-small cell lung cancers; SCLC: small-cell lung cancer.

**Supplementary Table S7. Cumulative lung cancer mortality and screening-related hazard ratios in current vs. former smokers, for deaths with corresponding LC diagnosis within maximally 6 years after scheduled final screening (total n = 786), by sex, histology and follow-up time after final screening; ITALUNG-LUSI and NLST combined.**

| **Years of follow-up for mortality, since the final scheduled screening** | **Males + Females** | | | | **Males** | | | | **Females** | | | |
| --- | --- | --- | --- | --- | --- | --- | --- | --- | --- | --- | --- | --- |
|  | **Screening**  **(*n* =15,168)** | **Control**  **(*n* = 15,154)** | **Δ** | **HR**  **(95% CI) ^a^** | **Screening**  **(*n =* 8,741)** | **Control**  **(*n =* 8,840)** | **Δ** | **HR**  **(95% CI) ^a^** | **Screening**  **(*n* = 6,427)** | **Control**  **(*n =* 6,350)** | **Δ** | **HR**  **(95% CI) ^a^** |
| **CURRENT SMOKERS** | | | | | | | | | | | | |
| ***All sub-types ^b^*** | | | | | | | | | | | | |
| **2** | 179 | 210 | -31 | 0.85  (0.70-1.04) | 129 | 134 | -5 | 0.96  (0.75-1.22) | 50 | 76 | -26 | **0.65**  **(0.46-0.93)** |
| **4** | 286 | 376 | -90 | **0.76**  **(0.65-0.89)** | 198 | 230 | -32 | 0.86  (0.71-1.04) | 88 | 146 | -58 | **0.61**  **(0.47-0.79)** |
| **6** | 355 | 431 | -76 | **0.82**  **(0.71-0.94)** | 237 | 260 | -23 | 0.91  (0.77-1.09) | 118 | 171 | -53 | **0.68**  **(0.54-0.86)** |
| ***Non-BAC LUAD ^c^*** | | | | | | | | | | | | |
| **2** | 56 | 59 | -3 | 0.92  (0.64-1.32) | 41 | 33 | +8 | 1.18  (0.75-1.87) | 15 | 26 | -11 | 0.57  (0.30-1.08) |
| **4** | 85 | 110 | -25 | 0.77  (0.58-1.02) | 54 | 62 | -8 | 0.89  (0.62-1.28) | 31 | 48 | -17 | **0.62**  **(0.40-0.97)** |
| **6** | 106 | 132 | -26 | 0.80  (0.62-1.03) | 69 | 73 | -4 | 0.95  (0.68-1.31) | 37 | 59 | -22 | **0.62**  **(0.41-0.93)** |
| ***LUSC ^d^*** | | | | | | | | | | | | |
| **2** | 42 | 38 | +4 | 1.11  (0.71-1.72) | 31 | 30 | +1 | 1.04  (0.63-1.72) | 11 | 8 | +3 | 1.36  (0.55-3.38) |
| **4** | 62 | 72 | -10 | 0.86  (0.61-1.21) | 47 | 57 | -10 | 0.81  (0.55-1.19) | 15 | 15 | 0 | 1.05  (0.52-2.13) |
| **6** | 76 | 81 | -5 | 0.93  (0.68-1.28) | 55 | 65 | -10 | 0.85  (0.59-1.21) | 21 | 16 | +5 | 1.29  (0.68-2.48) |
| ***Other NSCLC ^e^*** | | | | | | | | | | | | |
| **2** | 31 | 52 | -21 | **0.60**  **(0.38-0.93)** | 21 | 35 | -14 | 0.60  (0.35-1.04) | 10 | 17 | -7 | 0.58  (0.27-1.27) |
| **4** | 45 | 87 | -42 | **0.52**  **(0.36-0.74)** | 31 | 53 | -22 | **0.59**  **(0.38-0.91)** | 14 | 34 | -20 | **0.41**  **(0.22-0.76)** |
| **6** | 57 | 90 | -33 | **0.63**  **(0.45-0.88)** | 38 | 54 | -16 | 0.71  (0.47-1.07) | 19 | 36 | -17 | **0.52**  **(0.30-0.91)** |
| ***SCLC ^f^*** | | | | | | | | | | | | |
| **2** | 45 | 52 | -7 | 0.88  (0.59-1.31) | 32 | 30 | +2 | 1.10  (0.67-1.81) | 13 | 22 | -9 | 0.59  (0.30-1.16) |
| **4** | 76 | 89 | -13 | 0.85  (0.63-1.15) | 52 | 50 | +2 | 1.04  (0.71-1.53) | 24 | 39 | -15 | 0.61  (0.37-1.01) |
| **6** | 90 | 103 | -13 | 0.87  (0.65-1.15) | 59 | 54 | +5 | 1.09  (0.76-1.58) | 31 | 49 | -18 | **0.62**  **(0.40-0.98)** |
| **FORMER SMOKERS** | | | | | | | | | | | | |
| ***All sub-types ^b^*** | | | | | | | | | | | | |
| **2** | 92 | 113 | -21 | 0.81  (0.62-1.07) | 66 | 75 | -9 | 0.86  (0.62-1.21) | 26 | 38 | -12 | 0.71  (0.44-1.17) |
| **4** | 157 | 181 | -24 | 0.85  (0.69-1.05) | 111 | 122 | -11 | 0.89  (0.69-1.16) | 46 | 59 | -13 | 0.76  (0.52-1.11) |
| **6** | 205 | 217 | -12 | 0.94  (0.77-1.13) | 143 | 146 | -3 | 0.96  (0.76-1.21) | 62 | 71 | -9 | 0.88  (0.62-1.23) |
| ***Non-BAC LUAD ^c^*** | | | | | | | | | | | | |
| **2** | 28 | 49 | -21 | **0.57**  **(0.36-0.90)** | 21 | 34 | -13 | 0.61  (0.35-1.05) | 7 | 15 | -8 | 0.47  (0.19-1.15) |
| **4** | 50 | 77 | -27 | **0.63**  **(0.45-0.91)** | 34 | 57 | -23 | **0.59**  **(0.38-0.90)** | 16 | 20 | -4 | 0.77  (0.40-1.47) |
| **6** | 73 | 91 | -18 | 0.80  (0.58-1.08) | 49 | 67 | -18 | 0.72  (0.50-1.04) | 24 | 24 | 0 | 1.00  (0.57-1.77) |
| ***LUSC ^d^*** | | | | | | | | | | | | |
| **2** | 23 | 11 | +12 | **2.18**  **(1.03-4.61)** | 18 | 7 | +11 | **2.79**  **(1.10-7.08)** | 5 | 4 | +1 | 1.25  (0.34-4.66) |
| **4** | 40 | 20 | +20 | **1.89**  **(1.11-3.20)** | 33 | 14 | +19 | **2.32**  **(1.24-4.33)** | 7 | 6 | +1 | 1.01  (0.35-2.87) |
| **6** | 47 | 28 | +19 | **1.67**  **(1.04-2.66)** | 37 | 18 | +19 | **2.02**  **(1.15-3.56)** | 10 | 10 | 0 | 1.09  (0.42-2.42) |
| ***Other NSCLC ^e^*** | | | | | | | | | | | | |
| **2** | 17 | 22 | -5 | 0.78  (0.42-1.44) | 8 | 13 | -5 | 0.56  (0.24-1.34) | 9 | 9 | 0 | 1.11  (0.45-2.74) |
| **4** | 28 | 36 | -8 | 0.77  (0.47-1.26) | 15 | 22 | -7 | 0.67  (0.35-1.29) | 13 | 14 | -1 | 0.93  (0.44-1.98) |
| **6** | 34 | 46 | -12 | 0.73  (0.47-1.14) | 18 | 28 | -10 | 0.63  (0.35-1.14) | 16 | 18 | -2 | 0.89  (0.45-1.74) |
| ***SCLC ^f^*** | | | | | | | | | | | | |
| **2** | 18 | 25 | -7 | 0.74  (0.40-1.37) | 14 | 18 | -4 | 0.81  (0.40-1.64) | 4 | 7 | -3 | 0.57  (0.17-1.96) |
| **4** | 26 | 36 | -10 | 0.71  (0.43-1.18) | 19 | 22 | -3 | 0.85  (0.46-1.57) | 7 | 14 | -7 | 0.50  (0.20-1.24) |
| **6** | 36 | 40 | -4 | 0.89  (0.57-1.40) | 27 | 26 | +1 | 1.02  (0.60-1.75) | 9 | 14 | -5 | 0.64  (0.28-1.49) |

HR was estimated using the proportional hazards model. Bold figures indicate hazard rates that significantly differ from 1.00 (*P* < 0.05).

^a^ Adjusted by age.

^b^ All subtypes: adenocarcinomas without BAC: ICD-O-3 = 8140, 8230, 8255, 8260, 8310, 8480, 8490, 8550, 8560; squamous cell carcinoma: ICD-O-3 = 8052, 8070, 8071, 8072, 8074, 8076, 8078, 8083; other non-small cell carcinoma: ICD-O-3 = 8010,8012, 8013, 8020, 8021, 8022, 8032, 8033, 8046, 8050, 8980; small cell carcinoma: ICD-O-3 = 8041, 8042, 8044, 8045; lepidic/non-mucinous bronchiolo-alveolar carcinoma (BAC): ICD-O-3 = 8250, 8252; mucinous bronchiolo-alveolar carcinoma (BAC): ICD-O-3 = 8253, 8254; other neuroendocrine: ICD-O-3 = 8240, 8246, 8249 and unclassified tumors: ICD-O-3 = 8000, 8001.

^c^ Adenocarcinomas without BAC: ICD-O-3 = 8140, 8230, 8255, 8260, 8310, 8480, 8490, 8550, 8560.

^d^ Squamous cell carcinoma: ICD-O-3 = 8052, 8070, 8071, 8072, 8074, 8076, 8078, 8083.

^e^ Other non-small cell carcinoma: ICD-O-3 = 8010,8012, 8013, 8020, 8021, 8022, 8032, 8033, 8046, 8050, 8980.

^f^ Small cell carcinoma: ICD-O-3 = 8041, 8042, 8044, 8045

Abbreviations: LUSI: Lung Cancer Screening Intervention study; ITALUNG: Italian Lung Study; NLST: National Lung Screening Trial; HR: hazard ratio; CI: confidence interval; Δ: Difference between screening and control arm; BAC: bronchiolo-alveolar carcinoma; ICD-O-3: International Classification of Diseases for Oncology, Third edition; LUAD: lung adenocarcinoma; LUSC: lung squamous cell carcinomas; NSCLC: non-small cell lung cancers; SCLC: small-cell lung cancer.

**Supplementary Table S8**. **Heterogeneity test for the effect of LDCT screening on overall and histology-specific LC mortality between women and men, accounting for baseline smoking status.**

| **Histology** | **HR (95% CI)** | ***P*** |
| --- | --- | --- |
| ***All sub-types*** | | |
| Age | **1.10 (1.09 – 1.12)** | **<0.001** |
| Arm (screening) | **0.71 (0.58 – 0.87)** | **0.001** |
| Arm (screening) ^x^ Smoking (former) | 1.13 (0.89 – 1.43) | 0.319 |
| Arm (screening) ^x^ Sex (male) | 1.25 (0.99 – 1.59) | 0.062 |
| ***Non-BAC adenocarcinomas (non-BAC LUAD)*** | | |
| Age | **1.09 (1.07 – 1.11)** | **<0.001** |
| Arm (screening) | 0.73 (0.51 – 1.05) | 0.094 |
| Arm (screening) ^x^ Smoking (former) | 0.99 (0.66 – 1.47) | 0.953 |
| Arm (screening) ^x^ Sex (male) | 1.15 (0.76 – 1.73) | 0.512 |
| ***Lung squamous cell carcinomas (LUSC)*** | | |
| Age | **1.10 (1.08 – 1.13)** | **<0.001** |
| Arm (screening) | 1.00 (0.58 – 1.73) | 0.994 |
| Arm (screening) ^x^ Smoking (former) | **1.78 (1.01 – 3.13)** | **0.045** |
| Arm (screening) ^x^ Sex (male) | 0.91 (0.50 – 1.66) | 0.761 |
| ***Other non-small cell lung carcinomas (other NSCLC)*** | | |
| Age | **1.10 (1.07 – 1.13)** | **<0.001** |
| Arm (screening) | **0.61 (0.39 – 0.98)** | **0.039** |
| Arm (screening) ^x^ Smoking (former) | 1.16 (0.66 – 2.01) | 0.608 |
| Arm (screening) ^x^ Sex (male) | 1.05 (0.61 – 1.81) | 0.861 |
| ***Small cell lung carcinomas (SCLC)*** | | |
| Age | **1.10 (1.08 – 1.13)** | **<0.001** |
| Arm (screening) | **0.63 (0.41 – 0.96)** | **0.031** |
| Arm (screening) ^x^ Smoking (former) | 1.00 (0.59 – 1.70) | 0.997 |
| Arm (screening) ^x^ Sex (male) | **1.70 (1.03 – 2.80)** | **0.038** |

HR was estimated using the proportional hazards model. Bold figures indicate hazard rates that significantly differ from 1.00 (*P* < 0.05). Abbreviations: HR: hazard ratio; CI: confidence interval; Δ: Difference between screening and control arm; BAC: bronchiolo-alveolar carcinoma; LUAD: lung adenocarcinoma; LUSC: lung squamous cell carcinomas; NSCLC: non-small cell lung cancers; SCLC: small-cell lung cancer.
